# Long-lasting geroprotection from brief rapamycin treatment in early adulthood by persistently increased intestinal autophagy

**DOI:** 10.1038/s43587-022-00278-w

**Published:** 2022-08-29

**Authors:** Paula Juricic, Yu-Xuan Lu, Thomas Leech, Lisa F. Drews, Jonathan Paulitz, Jiongming Lu, Tobias Nespital, Sina Azami, Jennifer C. Regan, Emilie Funk, Jenny Fröhlich, Sebastian Grönke, Linda Partridge

**Affiliations:** 1 Max Planck Institute for Biology of Ageing, Cologne, Germany; 2 Institute of Healthy Ageing, Department of Genetics, Evolution and Environment, University College London, London, UK; 3 Present Address: Lunaphore Technologies SA, Tolochenaz, Switzerland; 4 Present Address: Institute of Immunology and Infection Research, University of Edinburgh, Edinburgh, UK

**Keywords:** Autophagy, Cell signalling, Ageing

## Abstract

The licensed drug rapamycin has potential to be repurposed for geroprotection. A key challenge is to avoid adverse side effects from continuous dosing. Here we show that geroprotective effects of chronic rapamycin treatment can be obtained with a brief pulse of the drug in early adulthood in female *Drosophila* and mice. In *Drosophila*, a brief, early rapamycin treatment of adults extended lifespan and attenuated age-related decline in the intestine to the same degree as lifelong dosing. Lasting memory of earlier treatment was mediated by elevated autophagy in intestinal enterocytes, accompanied by increased levels of intestinal LManV and lysozyme. Brief elevation of autophagy in early adulthood itself induced a long-term increase in autophagy. In mice, a 3-month, early treatment also induced a memory effect, with maintenance similar to chronic treatment, of lysozyme distribution, Man2B1 level in intestinal crypts, Paneth cell architecture and gut barrier function, even 6 months after rapamycin was withdrawn.

## Abstract (web summary)

A key challenge for repurposing the licensed drug rapamycin for geroprotection is to avoid side effects from chronic dosing regimens. The authors show in model organisms that a brief administration of the drug early in adulthood has long-lasting beneficial effects that are similar to lifelong treatment.

## Main

The macrolide drug rapamycin inhibits TORC1 activity and can extend lifespan in model organisms, including mice^1–3^. In mice, rapamycin can delay several age-related diseases, such as cognitive decline^4^, spontaneous tumors^5^ and cardiovascular^6,7^ and immune^8^ dysfunction. However, chronic rapamycin administration can cause adverse effects, even with low doses^9,10^. Shortening treatment could potentially reduce negative effects. Short-term treatment in late life can extend lifespan in mice^3,11,12^ and enhance immune response in older people^13,14^. However, it is unknown whether the effects of late-life treatment are comparable to those of lifelong drug exposure, or whether brief treatment at younger ages is sufficient to gain the benefits of the chronic treatment.

**Fig. 1:**
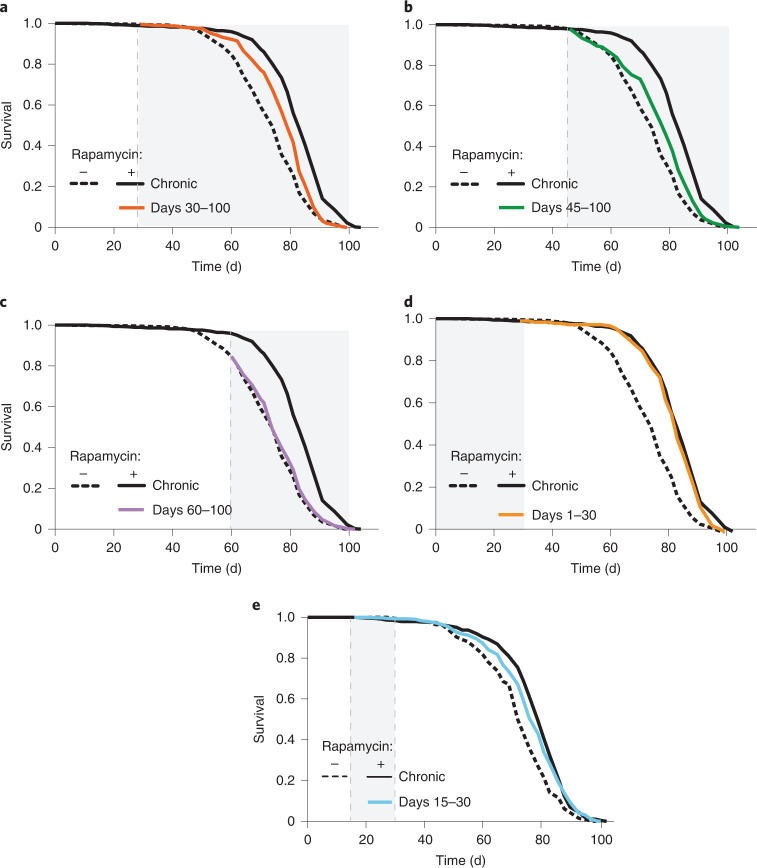
Lifespan response to rapamycin treatment declines with the age of onset of the treatment. **a**, Rapamycin treatment started on day 30 extended lifespan (*P* = 2.13 × 10^−6^) to a lesser degree than did lifelong treatment (*P* = 1.04 × 10^−13^; Supplementary Table 2). **b**,**c**, Rapamycin treatment started on day 45 modestly extended lifespan (**b**; *P* = 0.0003; Supplementary Table 1), whereas treatment started on day 60 (**c**) had no lifespan-extending effect (*P* = 0.256; Supplementary Table 1). **d**, Rapamycin treatment from day 1–30 extended lifespan (*P* = 2.13 × 10^−6^) as much as did chronic treatment (*P* = 0.09; Supplementary Table 2). **e**, Treatment from days 15–30 extended lifespan slightly less than did chronic treatment (d15–30 versus control, *P* = 7.58 × 10^−7^; d15–30 versus chronic rapamycin, *P* = 0.19; Supplementary Table 3). Experiments in Extended Data Fig. 1a–d and Figs. 1e and 2a were run in parallel, thus lifespan data of the control flies are the same. *N* = 400 flies per condition. Source data

To assess the efficacy of late-onset rapamycin treatment, we treated *Drosophila* females, which increased lifespan in response to rapamycin treatment substantially greater than did treatment in males^2,15^, at different ages and for varying durations. Treatments starting later in life, on day 30 or day 45, extended lifespan, consistent with previous findings in mice^3,11,12^, but less than did lifelong treatment (Fig. 1a,b and Supplementary Tables 1 and 2). Very late-onset rapamycin treatment from day 60, when survival was already decreased to ~80%, did not increase lifespan (Fig. 1c and Supplementary Table 2). Thus, later-onset rapamycin treatment produced progressively smaller extensions of lifespan.

**Fig. 2:**
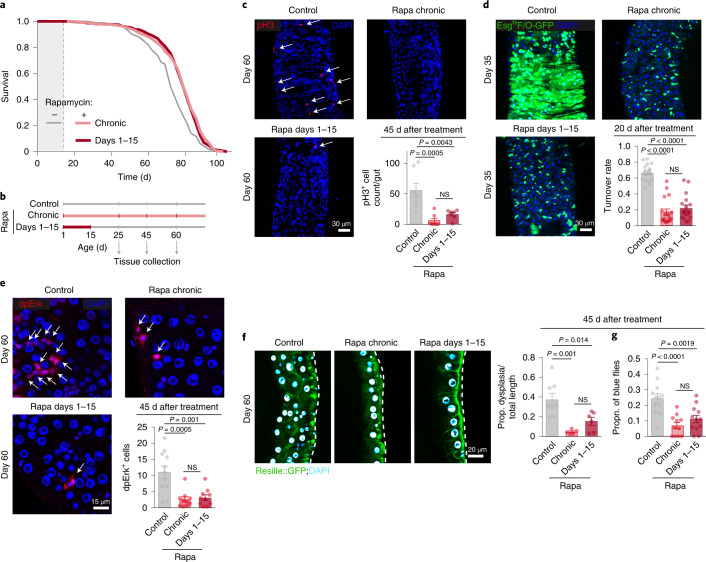
Brief rapamycin treatment early in adulthood extends lifespan and preserves intestinal function as much as does chronic treatment. **a**, Lifespan of flies chronically or in days 1–15 treated with rapamycin (*n* = 400 per condition; Supplementary Table 3). **b**, Experimental design. **c**, The number of pH3^+^ cells (arrows) in the gut 45 d after the short-term rapamycin treatment was withdrawn (*n* = 7–8). **d**, Midgut turnover rate, as assessed with the esg^ts^F/O system 20 d after treatment (*n* = 15–18). **e**, The number of dpErk^+^ cells 45 d after rapamycin treatment (*n* = 10–11). **f**, Intestinal dysplasia in the gut R2 region of flies carrying epithelial marker Resille-GFP 45 d after short-term rapamycin treatment was terminated (*n* = 6–8). **g**, Intestinal barrier function in flies treated with rapamycin chronically or in days 1–15. Data are the mean ± s.e.m. One-way analysis of variance (ANOVA), Bonferroni’s post hoc test. NS, not significant. Source data

In sharp contrast, rapamycin treatment instigated early in adulthood on day 3 following eclosion and 2 d of mating (termed ‘day 1’), for just 30 d, extended lifespan as much as did lifelong dosing (Fig. 1d and Supplementary Table 3). Treatment from days 15–30 increased lifespan, but less than did chronic treatment (Fig. 1e and Supplementary Table 3). Remarkably, rapamycin in only the first 15 d of adult life recapitulated the full lifespan extension achieved by chronic treatment (Fig. 2a and Supplementary Table 3), a phenomenon we termed ‘rapamycin memory’.

**Extended Data Fig. 1:**
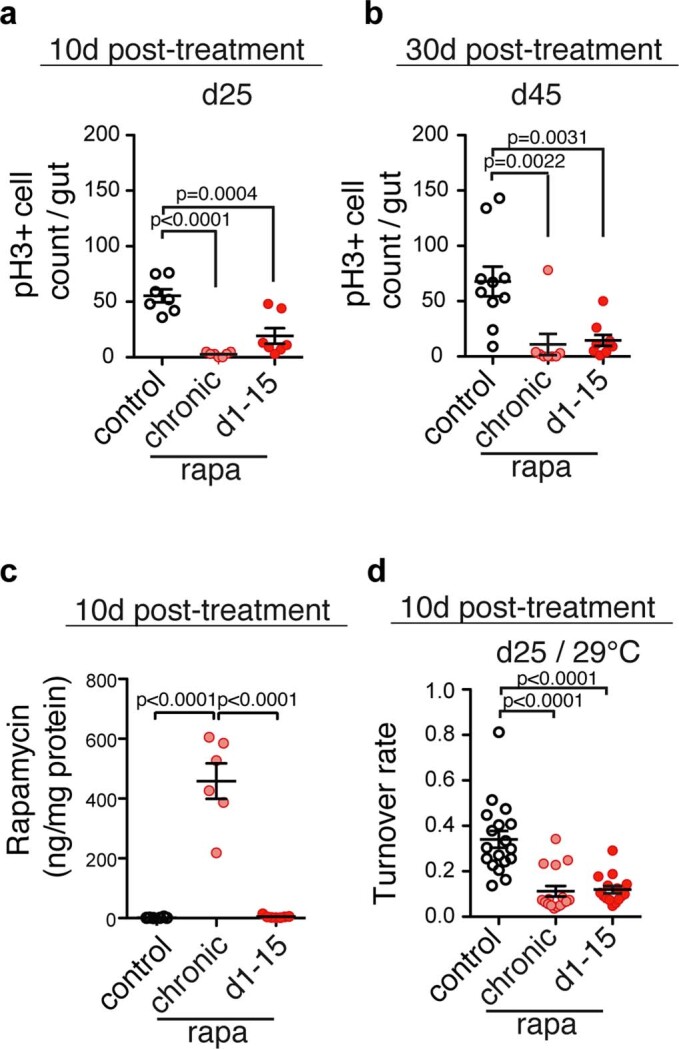
Brief rapamycin treatment inhibits ISC division and epithelial turnover rate to the late stages of life. **a-b**, Rapamycin treatment from day 1-15 reduced pH3+ cell number 10 (a, n = 7) and 30 (b, n = 8-10) days post-treatment to the same degree as rapamycin chronic exposure. **c**, Rapamycin concentration was increased in the guts of 25-day-old flies chronically treated with rapamycin but was absent from the intestine 10-days after rapamycin treatment was terminated. n = 6-7. **d**, Turnover of intestinal epithelium was reduced 10 days after the treatment termination as assessed by *esg*^*ts*^ *F/O s*ystem activation (at 29 °C). Data are mean ± s.e.m. One-way ANOVA followed by Bonferroni’s post-test.

Rapamycin increases lifespan mainly in female *Drosophila*^2^. The number of dividing intestinal stem cells (ISCs) increases with age in female flies, to restore damaged parts of the intestinal epithelium, driving intestinal dysplasia later in life^16^. Thus, we hypothesized that short-term rapamycin might permanently alter ISC activity. As previously reported^17^, chronic rapamycin treatment reduced pH3^+^ cell number (Fig. 2c), a marker for dividing cells^18^. Strikingly, the number of pH3^+^ cells of flies treated with rapamycin only during days 1–15 remained as low as in flies treated chronically, even 10, 30 and 45 d after treatment (Fig. 2b,c and Extended Data Fig. 1a,b). Mass spectrometry confirmed that rapamycin concentration was reduced to the level of control flies 10 d after rapamycin treatment on days 1–15 was ended (Extended Data Fig. 1c). The ISCs thus remained fully quiescent long after rapamycin had been cleared.

We next assessed the turnover rate of the intestinal epithelium using the *esg*^*ts*^ *F/O system (esg-Gal4; tubGal80*^*ts*^ *Act* > *CD2* > *Gal4 UAS-Flp UAS-GFP)*^19^, where activation by a temperature shift to 29 °C marks ISCs and their progenitor cells with GFP. Under standard conditions, the epithelial turnover rate in *Drosophila* is 14 d. Temperature increase shortens lifespan, so we measured turnover rate 10 and 20 d after treatment. Most of the control midgut epithelium was replaced by GFP-positive cells after 10 d (Extended Data Fig. 1d) and 20 d (Fig. 2d) of system activation. Chronic and day 1–15 rapamycin treatment reduced the number of GFP-positive cells 10 and 20 d after the switch to the same extent (Fig. 2d and Extended Data Fig. 1d). Brief, early rapamycin exposure thus reduced turnover of the intestinal epithelium as much as chronic treatment, and the cells previously treated with rapamycin remained in the gut until advanced age.

Staining with diphosphorylated Erk (dpErk), a specific readout for signal that damaged and apoptotic enterocytes send to the ISCs for replacement^20^, revealed that short-term rapamycin treatment reduced the number of apoptotic, dpErk-positive cells as much as did chronic treatment (Fig. 2e), suggesting increased enterocyte health. We therefore assessed if intestinal pathologies were reduced. Histology using the epithelial marker Resille-GFP revealed that dysplastic regions were widespread throughout the guts of aging control flies (Fig. 2f). Flies treated chronically with rapamycin had significantly fewer dysplastic lesions at day 60. Interestingly, the proportion of dysplastic regions remained reduced 45 d after short-term rapamycin treatment was withdrawn, to the same degree as that seen with chronic treatment (Fig. 2f). Because lifespan is directly linked to gut barrier function, and loss of septate junction proteins disrupts gut integrity^21^, we measured the effect of brief rapamycin treatment on gut barrier function. Intestinal integrity, as measured by a blue dye leakage assay, was preserved by rapamycin treatment, and remained fully protected even 45 d after rapamycin was withdrawn (Fig. 2g). Taken together, these results indicate that brief, early-life rapamycin exposure exerted long-lasting protective effects on the intestine by reducing turnover of the epithelium, and preventing age-related increase in ISC proliferation, dysplasia and loss of intestinal barrier function.

**Extended Data Fig. 2:**
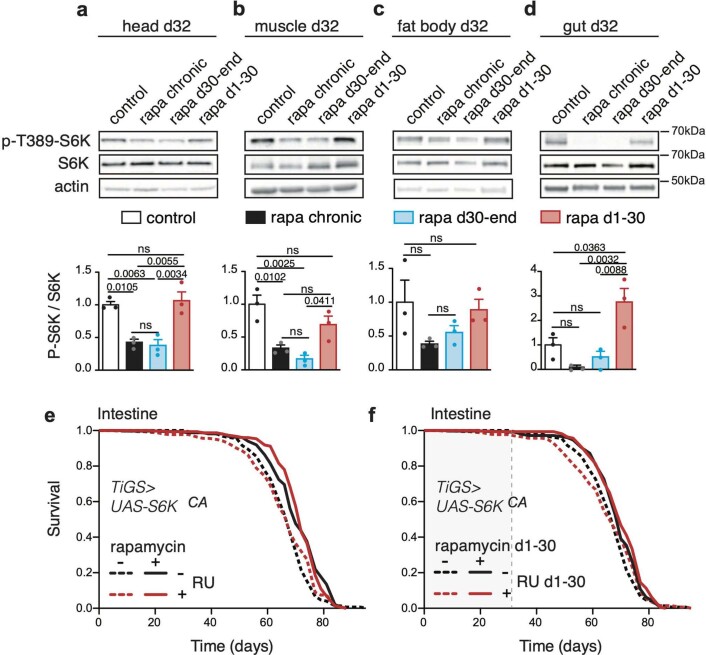
Effect of short-term rapamycin treatment on tissue-specific TORC1 activity and lifespan of flies over-expressing constitutively active S6K in the gut. **a-d**, Rapamycin treatment initiated on day 30 down-regulated TORC1 activity within 48 h and terminating rapamycin treatment on day 30 de-repressed TORC1 activity back to the levels of control flies within 48 h in heads, muscle, fat body and gut. Data are mean ± s.e.m. One-way ANOVA; Tukey’s multiple comparison test. N = 3 biological replicates, each consisting of 15 flies. **e-f**, Chronic (p = 9.95 ×10-05) and short-term (p = 0.029) rapamycin treatment extended lifespan of control flies and of flies over-expressing constitutively active S6K in the gut (chronic: p = 0.002, short-term treatment: p = 0.02, see also Supplementary Table 4). N = 200 flies per experimental group. Log-rank test and CPH analysis.

Persisting effects of brief rapamycin treatment could indicate a persistent inhibition of TORC1 activity. S6K is a direct target of TORC1 and reduced phosphorylation of S6K is required for extension of lifespan by rapamycin^2^. Rapamycin treatment instigated later in life, on day 30, reduced TORC1 activity within 48 h to the same level as chronic treatment in head, muscle, fat body and gut (Extended Data Fig. 2a–d). In contrast to lifespan, terminating rapamycin treatment on day 30 de-repressed TORC1 activity to the level of control flies in all four tissues (Extended Data Fig. 2a–d). In accordance with the absence of a ‘memory effect’ for intestinal S6K phosphorylation, overexpression of constitutively active S6K in the gut did not abolish lifespan extension by chronic or short-term rapamycin treatment (Extended Data Fig. 2e,f and Supplementary Table 4). Thus, TORC1 activity responded acutely to rapamycin, and events downstream of TORC1 other than reduced activity of S6K in the intestine, induced the ‘rapamycin memory’ effects.

**Fig. 3:**
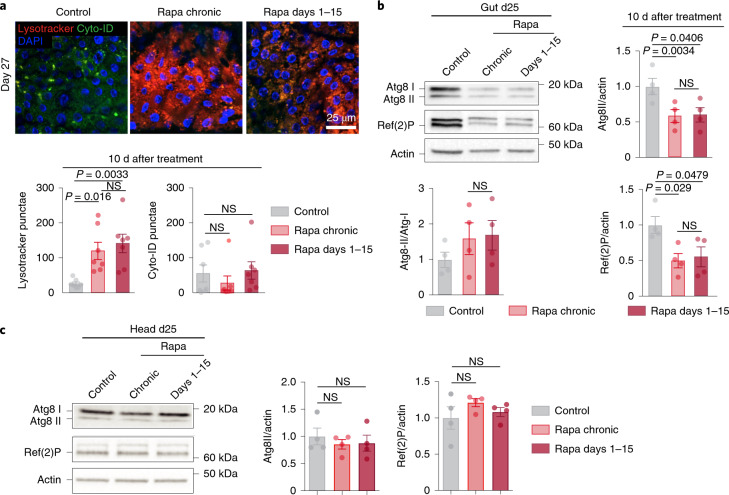
Short-term rapamycin treatment induces lasting autophagy activation. **a**, Number of punctae stained by LysoTracker and Cyto-ID in 25-day-old flies treated with rapamycin either chronically or in days 1–15 (*n* = 7). **b**,**c**, Immunoblot of autophagy-related proteins, Atg8-I, Atg8-II and Ref-2-P in the fly gut (**b**) and the head (**c**) on day 25, 10 d after rapamycin treatment. Data are the mean ± s.e.m. One-way ANOVA; Bonferroni’s multiple-comparison test. *n* = 4 biological replicates, each consisting of ten flies. Source data

**Extended Data Fig. 3:**
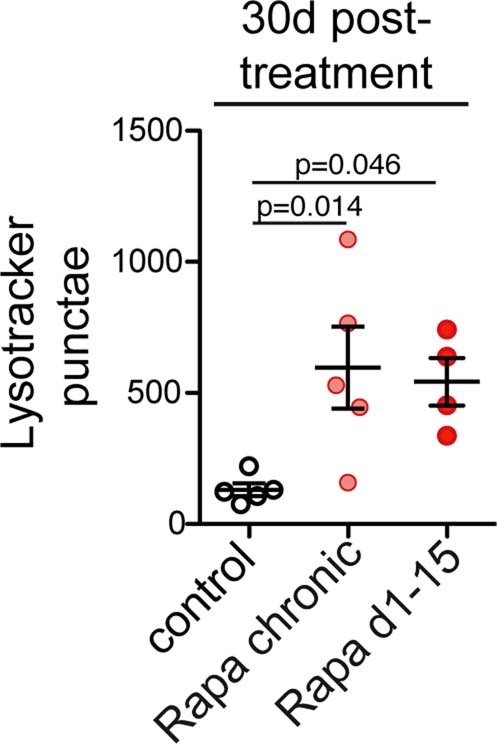
Effect of brief rapamycin treatment on the number of lysotracker + punctae in the gut 30-days post-treatment. Rapamycin induced lysotracker-stained punctae in the gut of 45-days old flies, and these stayed induced 30-days post-treatment. N = 4-5. Data are mean ± s.e.m. One-way ANOVA; Bonferroni’s multiple comparison test.

Increased autophagy is also a downstream effector of TORC1 and is required for lifespan extension by rapamycin^2^. Persistently upregulated autophagy could therefore carry the ‘memory of rapamycin’. To assess autophagic flux, we performed co-staining with Cyto-ID and lysotracker dye. While Cyto-ID specifically labels autophagosomes, lysotracker stains autolysosomes, and an increased ratio of autolysosomes to autophagosomes indicates increased autophagic flux^22^. Chronic rapamycin treatment increased levels of autolysosomes, without altering the levels of autophagosomes, indicative of an increased autophagic flux (Fig. 3a). Strikingly, the number of LysoTracker-stained punctae remained fully elevated even 10 d (Fig. 3a) and 30 d (Extended Data Fig. 3) after rapamycin was withdrawn, with no change in Cyto-ID-positive punctae (Fig. 3a). Immunoblot analysis revealed that chronic treatment decreased the levels of intestinal, non-lipidated and lipidated forms of the Atg8 protein and the *Drosophila* p62 homolog Ref-2-P and these stayed low 10 d after the treatment from days 1–15 was withdrawn (Fig. 3b), indicative of persistently activated autophagy. However, rapamycin had no effect on Atg8 and Ref-2-P in heads (Fig. 3c), suggesting a tissue-specific response. Together, these results suggest that autophagy induced by brief rapamycin treatment stayed induced for a prolonged period after rapamycin was withdrawn, despite TORC1 activity being restored back to control levels within 48 h.

**Fig. 4:**
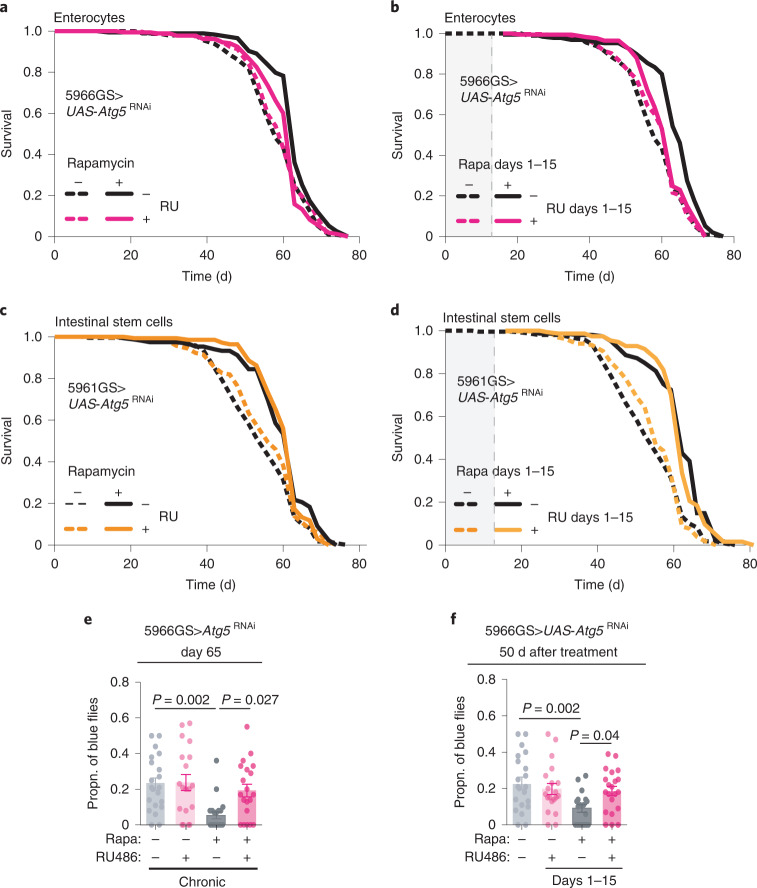
Enterocyte-specific autophagy induction mediates lifespan extension and gut barrier protection by short-term rapamycin treatment. **a**,**b**, Chronic (*P* = 1.53 × 10^−5^) and brief rapamycin (*P* = 8.8 × 10^−10^) treatment extended lifespan of control flies, but not of flies expressing RNAi against *Atg5* in enterocytes (chronic. *P* = 0.25; d1–15, *P* = 0.097; Supplementary Table 5). *n* = 400. **c**,**d**, Chronic (*P* = 3.4 × 10^−6^) and brief (*P* = 8.2 × 10^−13^) rapamycin treatment extended lifespan of control flies and flies with *Atg5*-RNAi specifically in ISCs (chronic, *P* = 0.001; d1–15, *P* = 5.4 × 10^−12^; Supplementary Table 6). *n* = 200. Log-rank test and Cox proportional hazard (CPH) analysis. **e**,**f**, Chronic and brief rapamycin treatment reduced the proportion of blue flies in the control group, but not in flies with enterocyte-specific *Atg5*-RNAi, on day 65. Rapamycin × genotype interaction (chronic, *P* = 0.057; d1–15, *P* = 0.020). *n* = 19–21 vials per condition with 20 flies in each vial. Data are the mean ± s.e.m. Two-way ANOVA followed by Bonferroni’s post hoc test. Source data

To test for a causal role of elevated autophagy in the intestine in the ‘rapamycin memory’, we abrogated it, both briefly and chronically, with double-stranded RNA-mediated interference (RNAi). We used inducible GeneSwitch drivers to drive expression of *Atg5-*RNAi in ISCs or enterocytes. Surprisingly, chronic and day 1–15 treatment with rapamycin both failed to increase lifespan of flies expressing *Atg5*-RNAi specifically in enterocytes (Fig. 4a,b and Supplementary Table 5), but not in ISCs (Fig. 4c,d and Supplementary Table 6). Furthermore, enterocyte-specific chronic and day 1–15 overexpression of *Atg5*-RNAi abrogated protection of gut barrier function by chronic and brief rapamycin exposure, respectively (Fig. 4e,f). Blocking the increase in autophagy in response to rapamycin in the enterocytes of the gut thus completely abolished the ‘rapamycin memory’ effect on both lifespan and intestinal integrity.

**Fig. 5:**
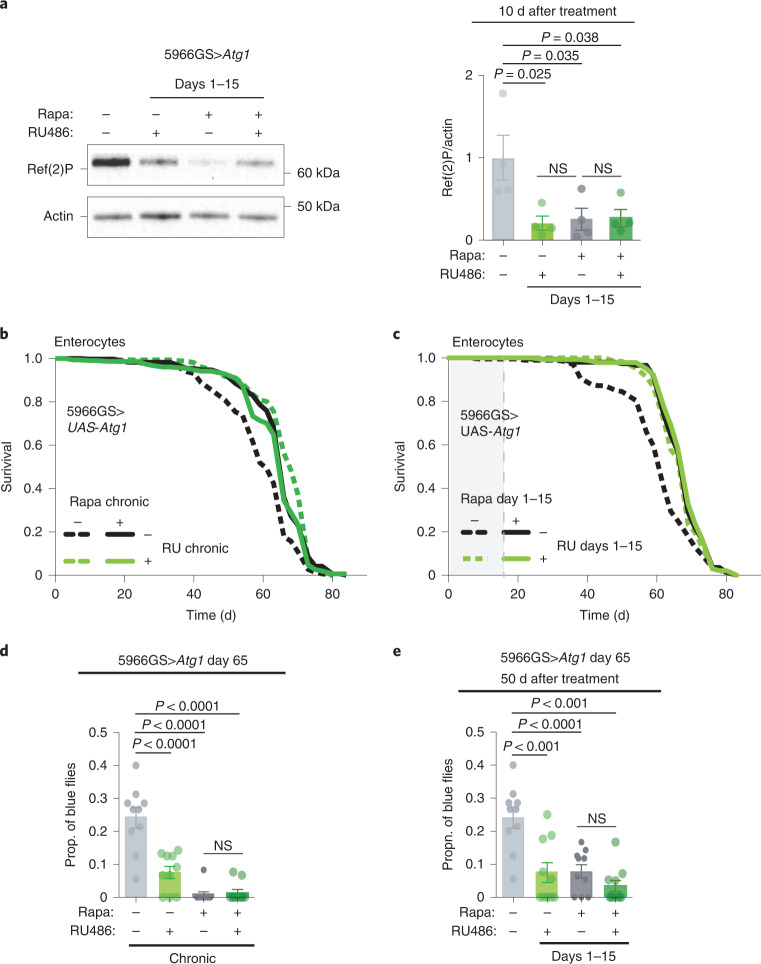
Short-term *Atg1* overexpression induces lasting autophagy activation and extends lifespan to the same degree as short rapamycin treatment. **a**, Immunoblot of intestinal Ref-2-P of flies treated with rapamycin from days 1–15 in combination with enterocyte-specific overexpression of *Atg1* in days 1–15, measured 10 d after treatment (*n* = 4). Genotype × rapamycin interaction (*P* = 0.03). **b**,**c**, Chronic (*P* = 3.6 × 10^−11^) and day 1–15 (*P* = 6.7 × 10^−5^) overexpression of *Atg1* specifically in enterocytes extended lifespan to the same degree as rapamycin (chronic, *P* = 0.50; d1–15, *P* = 0.69; Supplementary Table 7). *n* = 160–200. Log-rank test and CPH analysis. **d**,**e**, Chronic and day 1–15 overexpression of *Atg1* reduced the proportion of blue flies to the same degree as rapamycin treatment, on day 65. Rapamycin × genotype interaction for chronic overexpression (*P* = 0.02). *n* = 10 vials per condition with 20 flies per vial. Data are the mean ± s.e.m. Two-way ANOVA; Bonferroni’s multiple-comparison test.

To determine whether direct, genetic activation of autophagy was sufficient to mimic the ‘memory of rapamycin’ in the absence of the drug, we overexpressed *Atg1*, which induces autophagy in flies^23^. Interestingly, similarly to rapamycin short-term treatment, overexpression of *Atg1* in enterocytes from days 1–15 caused lasting downregulation of Ref-2-P 10 d after *Atg1* overexpression was terminated, while combining rapamycin with enterocyte-specific overexpression of *Atg1* from days 1–15 did not further reduce Ref-2-P levels (Fig. 5a). Furthermore, lifelong and day 1–15 enterocyte-specific overexpression of *Atg1* extended lifespan (Fig. 5b,c) and prevented age-related loss of intestinal integrity (Fig. 5d,e) as much as did chronic or brief rapamycin exposure, and the combination of *Atg1* overexpression and rapamycin did not further increase lifespan (Fig. 5b,c and Supplementary Table 7) nor improve gut barrier function (Fig. 5d,e). Thus, brief elevation of autophagy in enterocytes induces a memory identical to that from brief rapamycin treatment and mediates the ‘memory of rapamycin’ in increased autophagy, intestinal health and lifespan.

**Extended Data Fig. 4:**
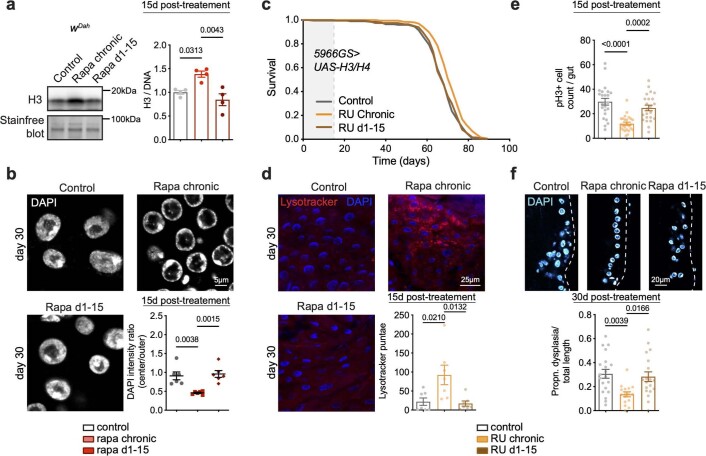
Increased expression of histones by chronic rapamycin treatment is not responsible for the effects of short-term rapamycin treatment on lifespan and gut health. **a**, Immunoblot of histone H3 in the fly gut on day 30, 15-days post-rapamycin-treatment (n = 4). **b**, The accumulation of chromatin at the nuclear envelope in ECs disappeared 15 days after d1-15 treatment was terminated (n = 6 guts). **c**, Chronic but not d1-15 over-expression of histones H3/H4 specifically in enterocytes extended lifespan (chronic: p = 7.39 ×10^-06^; d1-15 treatment: p = 0.60. n = 160-200 flies per experimental group. Log-rank test. **d**, Number of punctae stained by LysoTracker in 30-day old flies overexpressing H3/H4 specifically in enterocytes chronically or in days 1-15 (n = 7). **e**, The number of pH3+ cells in the gut 30 days after short-term rapamycin treatment was withdrawn (n = 23-25). **f**, Intestinal dysplasia in gut R2 region of flies 30-days after short-term rapamycin treatment was terminated (n = 16-20). Data are mean ± s.e.m. One-way ANOVA; Bonferroni’s multiple comparison test.

To test whether the recently reported rapamycin-mediated increase in histone expression^22^ underlies rapamycin memory, we investigated histone H3 expression after days 1–15 of rapamycin treatment and the effects of overexpression of H3/H4 during days 1–15 on autophagy, gut health and lifespan. As expected, H3 expression and accumulation of chromatin at the nuclear envelope were induced by chronic rapamycin treatment but were decreased back to control levels 15 d after treatment (Extended Data Fig. 4a,b). Moreover, although chronic overexpression of H3/H4 extended lifespan, decreased pH3^+^ cell count and intestinal dysplasia, and increased LysoTracker staining, these phenotypes showed no memory of the previous H3/H4 expression during days 1–15 (Extended Data Fig. 4c–f). These data suggest that, although increased histone expression mediates lifespan extension by chronic rapamycin treatment, this mechanism is distinct from the one that is responsible for the memory of short-term rapamycin treatment.

**Fig. 6:**
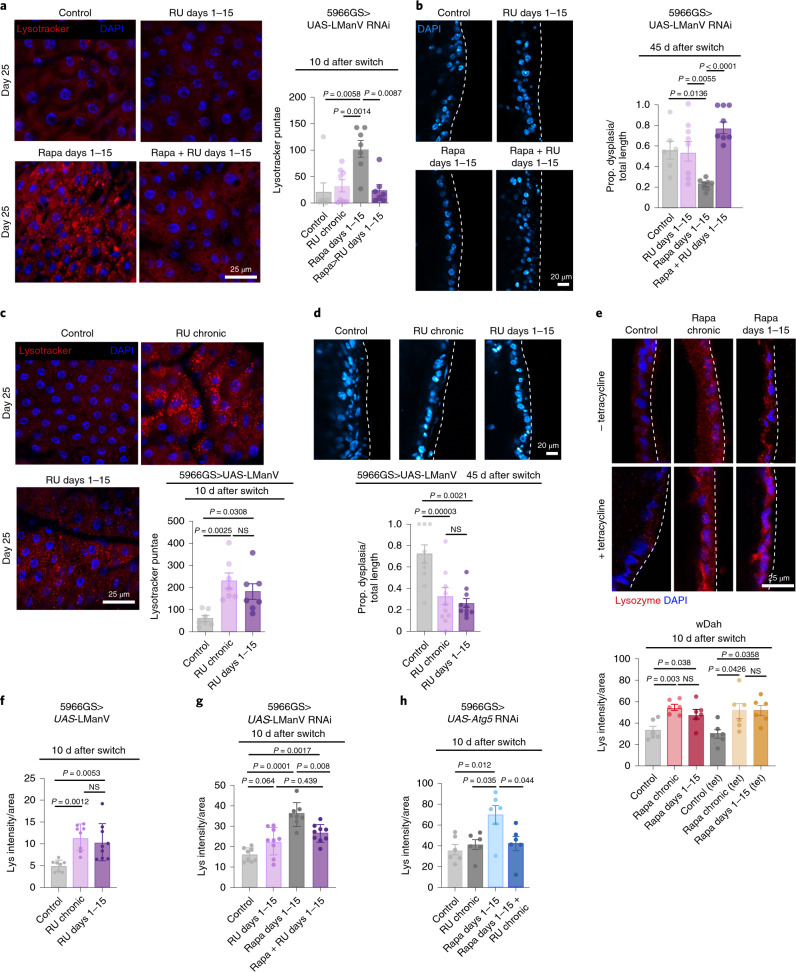
Persistent increase in LManV mediates the ‘memory of autophagy’ and reduced age-related gut pathology induced by short-term rapamycin treatment. **a**,**b**, Overexpression of RNAi against LManV in enterocytes in days 1–15 abolished the increase in LysoTracker staining (**a**) and reduction in gut pathology (**b**) induced by short-term rapamycin treatment (*n* = 7 flies). **c**,**d**, overexpression of LManV in enterocytes in days 1–15 increased LysoTracker staining (**c**) and reduced age-related gut pathology (**d**) to the same degree as chronic overexpression of LManV (*n* = 9–10 flies). **e**, Chronic and short-term rapamycin treatment increased intestinal lysozyme level irrespective of tetracycline treatment (*n* = 6 flies). **f**, Chronic and short-term overexpression of LManV increased lysozyme levels to the same degree (*n* = 8–9 flies). **g**, Overexpression of RNAi against LManV in enterocytes in days 1–15 abolished the increase in lysozyme level induced by rapamycin treatment in days 1–15 (*n* = 8–9 flies). **h**, Overexpression of RNAi against *Atg5* in enterocytes in days 1–15 abolished the increase in lysozyme level induced by rapamycin treatment in days 1–15 (*n* = 6 flies). Data are the mean ± s.e.m. One-way (**c**, **d** and **f**) and two-way (**a**, **b**, **e**, **g** and **h**) ANOVA; Bonferroni’s multiple-comparison test.

**Extended Data Fig. 5:**
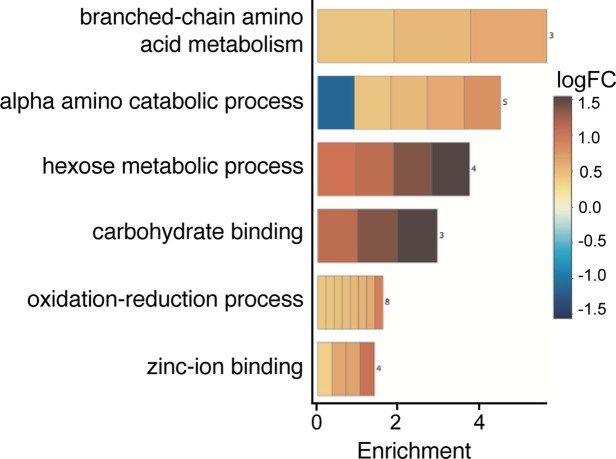
Rapamycin induces lasting changes in proteome of the gut. GO term analysis of proteins enriched by rapamycin treatment on day 25 that were also enriched 10 days after the drug was withdrawn.

**Extended Data Fig. 6:**
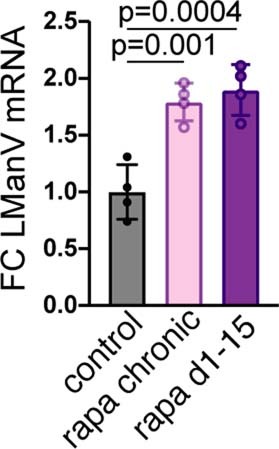
Intestinal LManV mRNA levels. Rapamycin treatment increased intestinal LManV mRNA, which stayed increased 10-days after the treatment was terminated. N = 4. Data are mean ± s.e.m. One-way ANOVA; Dunnett’s multiple comparison test.

To search for regulators of the ‘memory effect’ of rapamycin and elevated autophagy, we performed proteomics analysis. Gene Ontology (GO) term enrichment analysis of the proteins that were increased by rapamycin treatment on day 25 and that remained induced 10 d after the treatment revealed high enrichment in proteins involved in branched-chain amino acid (BCAA) and carbohydrate metabolism, in particular lysosomal mannosidases (Extended Data Fig. 5). We also found an increase in lysosomal alpha-mannosidase V (LManV) mRNA levels by quantitative PCR with reverse transcription (RT–qPCR; Extended Data Fig. 6). We therefore tested if knock-down of LManV abolished the ‘memory of rapamycin’. Indeed, knock-down of LManV blocked both the increase in LysoTracker-stained punctae by rapamycin treatment during days 1–15 (Fig. 6a) and the improved gut pathology mediated by short-term rapamycin treatment (Fig. 6b). To test if LManV activation was sufficient to mimic short-term rapamycin treatment, we overexpressed LManV during days 1–15, and found that it increased LysoTracker-stained punctae and reduced age-related gut pathologies to the same degree as chronic overexpression of LManV (Fig. 6c,d). Taken together, these findings suggest that the ‘memory of rapamycin’ in elevated autophagy and improved gut health is mediated through increased expression of LManV.

Recent studies showed that lysozyme-associated secretory autophagy plays a key role in gut health and pathogenesis in mammalian small intestine^24,25^. Secretory autophagy is an autophagy-based alternative secretion system that is activated in response to infection, and it is mediated by core autophagy proteins Atg5 and Atg16L1. Based on our data suggesting the importance of autophagy in the gut for the ‘memory of rapamycin’, we assessed whether levels of intestinal lysozyme, as a proxy for secretory autophagy, were affected by rapamycin treatment. We found that they were increased and remained fully so 10 d after the treatment was withdrawn. These responses to rapamycin were unaffected by tetracycline treatment (Fig. 6e), suggesting that the intestinal microbiota did not play a role. To investigate if LManV and autophagy were responsible for inducing increased lysozyme levels, we measured lysozyme levels in the intestines of flies overexpressing LManV, and found that both chronic and short-term overexpression increased lysozyme levels to the same degree. Knock-down of LManV by RNAi partially abolished increased lysozyme levels by rapamycin treatment in days 1–15 (Fig. 6f,g), while blocking autophagy by RNAi against *Atg5* abolished the increase in lysozyme levels induced by days 1–15 of rapamycin treatment (Fig. 6h). Together, these data suggest that autophagy and LManV mediate the rapamycin-induced increase in intestinal lysozyme levels.

**Extended Data Fig. 7:**
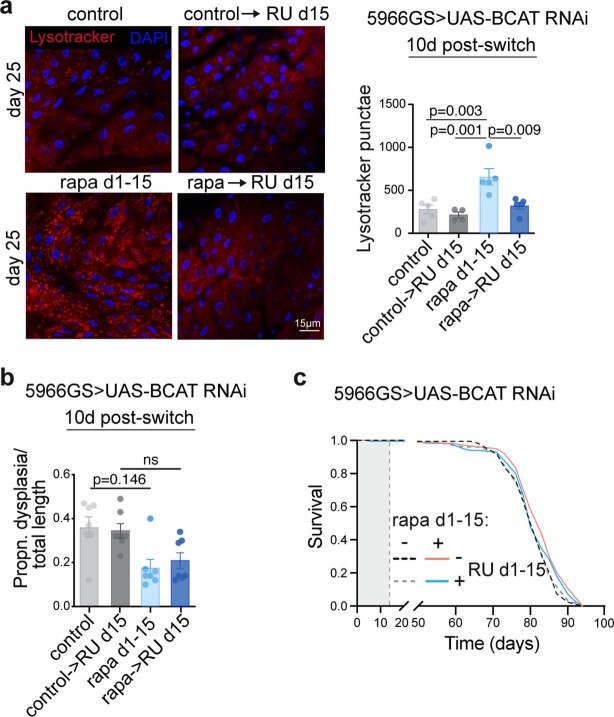
Persistent increase in BCAT mediates the ‘memory of autophagy’, and partially mediates gut pathology and lifespan induced by short-term rapamycin treatment. **a-b**, Number of LysoTracker stained punctae in intestines (**a**) and gut pathology (**b**) of flies treated with rapamycin from day 1-15 followed by over-expression of RNAi against BCAT (n = 5-7). Data are mean ± s.e.m. Two-way ANOVA; Bonferroni’s multiple comparison test. **c**, Brief rapamycin (p = 7.8 ×10^-4^) treatment extended lifespan of control flies, but not of flies expressing RNAi against BCAT in enterocytes (p = 0.14, see also Supplementary Table 9). Log-rank test. n = 180.

Branched-chain amino acid aminotransferase (BCAT) is one of the enzymes catabolizing the first step of BCAA degradation and we thus tested if knock-down of BCAT also abolished the ‘memory of rapamycin’. Expression of RNAi against BCAT in enterocytes from day 15 onwards blocked the increased number of LysoTracker-stained punctae (Extended Data Fig. 7a). Although there was a trend toward reduced intestinal dysplasia by rapamycin treatment, the effect was not significant (Extended Data Fig. 7b), and nor were the effects on intestinal dysplasia or lifespan (Extended Data Fig. 7b,c). Taken together, these findings suggest that BCAT contributes to the ‘memory of autophagy’ and further tests are needed to understand if BCAT mediates the effects of rapamycin on gut health and longevity.

**Fig. 7:**
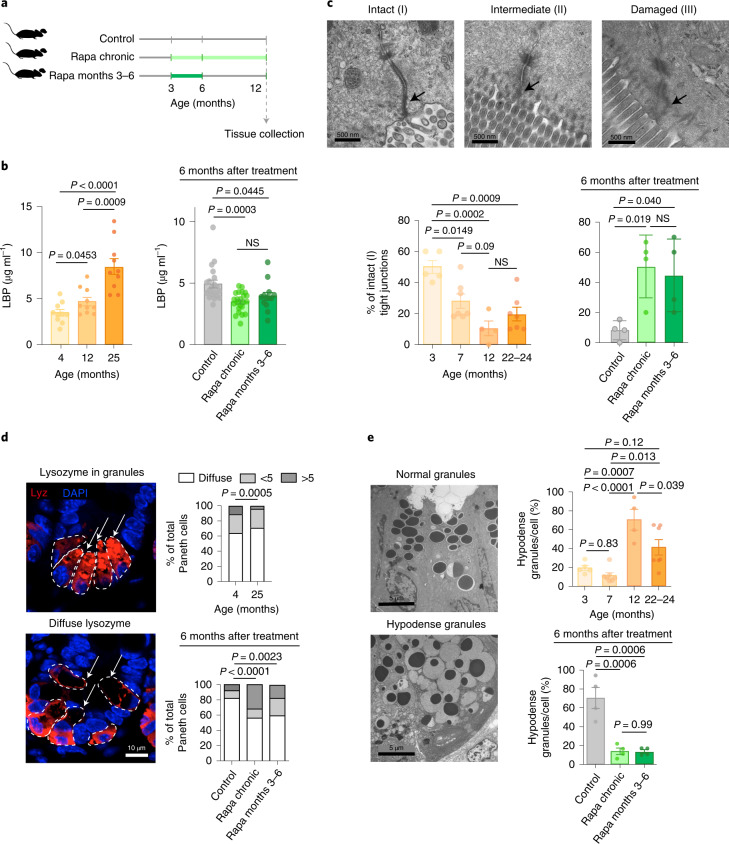
Short-term rapamycin exposure maintains gut barrier function and Paneth cell architecture to the same degree as lifelong treatment in mice. **a**, Experimental design. **b**, Plasma LBP levels during aging and 6 months after rapamycin treatment was terminated (*n* = 10–15 mice). **c**, TJ pathology score: I, narrow and electron-dense TJs; II, reduced electron density, but no dilations within TJs; III, low electron density and dilated TJs. Proportion of intact TJs during aging and 6 months post-rapamycin-treatment (*n* = 4–7 mice). **d**, The proportion of Paneth cells with diffuse lysozyme staining was increased in aged mice and rapamycin reduced the proportion of Paneth cells (arrows) with diffuse lysozyme staining, which remained reduced 6 months after treatment (*n* = 4). **e**, Proportion of hypodense Paneth cell granules in mouse jejunum during aging and 6 months after rapamycin treatment was withdrawn. *n* = 4. Data are the mean ± s.e.m (**b** and **e**) and s.d. (**c**; rapa treatment). One-way ANOVA; Bonferroni’s multiple-comparison test.

To assess if lasting benefits of a short-term rapamycin treatment are conserved between flies and mammals, we assessed the impact on intestinal permeability in mice (Fig. 7a), by measuring plasma lipopolysaccharide-binding protein (LBP) levels, a marker of bacterial translocation from intestine into circulation^26,27^. As we (Fig. 7b) and others^28^ showed that the age-related increase in gut permeability in rodents appears already in middle age, we treated mice with rapamycin chronically or from 3–6 months of age, and collected samples 6 months after the treatment was withdrawn, at 12 months of age (Fig. 7a). Strikingly, 6 months after rapamycin was withdrawn, plasma LBP levels were reduced to levels similar to those with chronic treatment, (Fig. 7b), suggesting that the long-lasting, beneficial effects of short-term rapamycin exposure on intestinal integrity is conserved in mammals.

**Extended Data Fig. 8:**
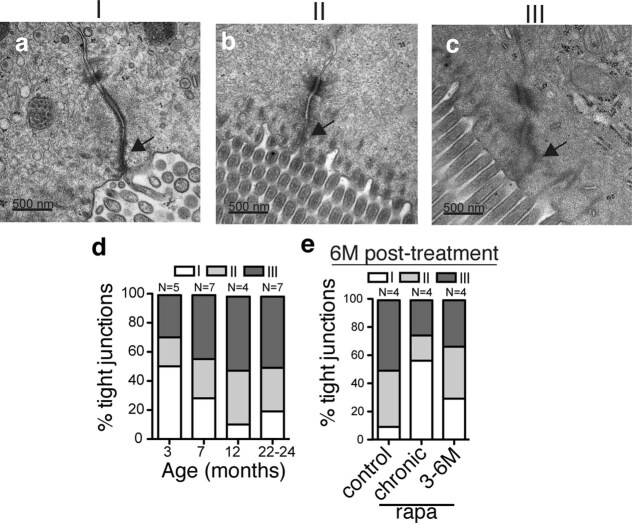
Effects of ageing and chronic and short-term rapamycin treatment on TJ. **a-c**, Tight junction pathology score: I - narrow and electron dense TJs (a); II - reduced electron density, but no dilations within TJs (b); III - low electron density and dilated TJs (c). **d**, Number of compromised TJs increased by 7 months of age compared to 3 months old mice (3 M v. 7 M: p = 0.0149; 3 M v. 12 M: p = 0.002; 3 M v. 22-24 M: p = 0.0009; 7 M v. 12 M: ns; 12 M v. 22-24 M: ns). **e**, Rapamycin reduced the percentage of compromised TJs (class II and III) in mouse jejunum (control v. rapa chronic: p = 0.0199; control v. rapa 3-6 M: 0.0405). Numbers of mice are indicated above each bar; at least 10 TJs per mouse were analyzed.

Increased gut permeability is associated with compromised tight junctions (TJs)^29^. Irregularities of TJ can be observed by electron microscopy as reduced electron density of the perijunctional ring^30^ and dilations within TJs^31^. We analyzed ultrastructure of TJs in jejunal villi. Intact TJs, which appeared as narrow and electron-dense structures, were classified as class I, narrow TJs with reduced electron density, but without dilations within the TJ as class II, and TJs that were both low in electron density and dilated as class III (Fig. 7c). In line with previously published data on gut permeability^28^, TJ quality declined during aging, with 7-month-old mice already showing a reduced proportion of intact TJs compared to 3-month-old mice (Fig. 7c). In accordance with plasma LBP results, rapamycin treatment increased the proportion of intact TJs, which remained increased 6 months after rapamycin withdrawal, further supporting the hypothesis that rapamycin protects age-related decline in intestinal integrity (Fig. 7c and Extended Data Fig. 8).

Paneth cells are specialized secretory cells that serve as a niche for ISCs^32^ and contain secretory granules filled with antimicrobial proteins, such as lysozyme, and rapamycin improves the Paneth cell function and their support of ISCs^33^. Lysozyme is normally efficiently packed in Paneth cell granules^24^. In 12-month-old control mice, we observed a notable proportion of Paneth cells with abnormal lysozyme distribution, which was diffuse in cells. Short-term rapamycin treatment increased the proportion of cells with lysozyme-packed granules and reduced those with a diffuse lysozyme signal (Fig. 7d). Transmission electron microscopy further showed that Paneth cell granule abnormalities, seen as loosely packed and hypodense granules that are a feature of dysfunction^24^, appeared already at 12 months of age (Fig. 7e). Remarkably, rapamycin treatment decreased the proportion of hypodense Paneth cell granules, which stayed decreased to the levels seen with chronic treatment 6 months after rapamycin treatment was withdrawn (Fig. 7e). Together, these data suggest that short-term rapamycin treatment abolished age-related Paneth cell abnormalities^24^.

**Fig. 8:**
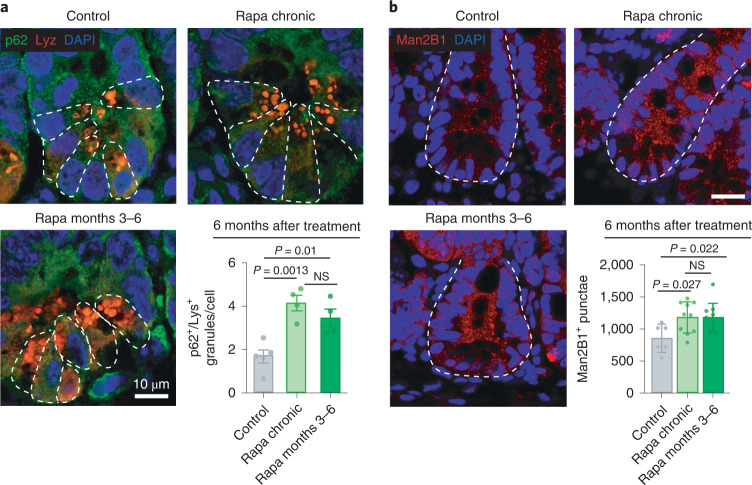
Short-term rapamycin exposure increases the number of granules positive for both lysozyme and p62 in Paneth cells and the number of Man2B1^+^ punctae in intestinal crypts to the same degree as lifelong treatment in mice. **a**, The number of lysozyme^+^/p62^+^ granules per Paneth cell was increased by rapamycin and remained increased 6 months after the treatment was withdrawn, at 12 months of age (*n* = 4 mice; at least ten Paneth cells per mouse were analyzed and the average value per mouse was used). White dashed lines denote Paneth cells. **b**, Number of Man2B1^+^ punctae was increased by rapamycin and remained increased 6 months after treatment, at 12 months of age (*n* = 6–10 mice). White dashed lines denote crypt units. Scale bar, 20 μm. Data are the mean ± s.e.m. One-way ANOVA; Bonferroni’s multiple-comparison test.

**Extended Data Fig. 9:**
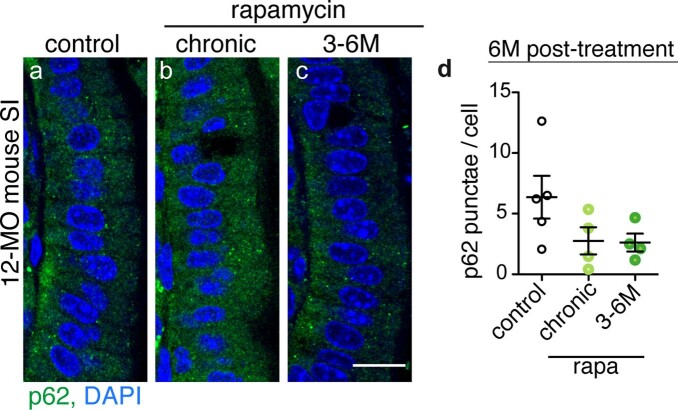
Rapamycin does not significantly affect the number of p62 punctae in the villi region of small intestine. **a-d**, p62 punctae in villi of small intestine. n = 4-5 mice, 50 or more cells in 5 different villi per mouse were analyzed. Data are mean ± s.e.m. One-way ANOVA, Bonferroni’s post-test. Scale is 15 μm.

Next, we assessed if the long-term elevation of autophagy by past rapamycin treatment is conserved in mice. Although chronic and 3–6 months rapamycin treatment did not significantly reduce the number of p62 punctae in the villi region, comprising enterocytes and goblet cells, there was a trend in the villi of 12-month-old rapamycin-treated mice (Extended Data Fig. 9). As autophagy is essential for proper Paneth cell function and secretion^24^, and upon autophagy activation autophagy-related proteins colocalize with Paneth cell granules^34^, we measured the number of granules positive for both lysozyme and p62. Chronic and 3–6 months rapamycin treatment increased the number of Paneth cell granules positive for both lysozyme and p62 assessed at 12 months of age (Fig. 8a), suggesting that autophagy in Paneth cells may play a key role in improving cell health in response to brief treatment, even 6 months after the drug is withdrawn.

As we showed that LManV is one of the mediators of rapamycin memory in *Drosophila*, and that it also mediates the rapamycin-induced increase in lysozyme levels in flies, we measured the levels of mannosidases in mouse gut. We observed that rapamycin increased the number of Man2B1-positive punctae in intestinal crypts, and these stayed increased 6 months after the treatment was withdrawn and to the same degree as with chronic treatment (Fig. 8b), in line with the fly data.

**Extended Data Fig. 10:**
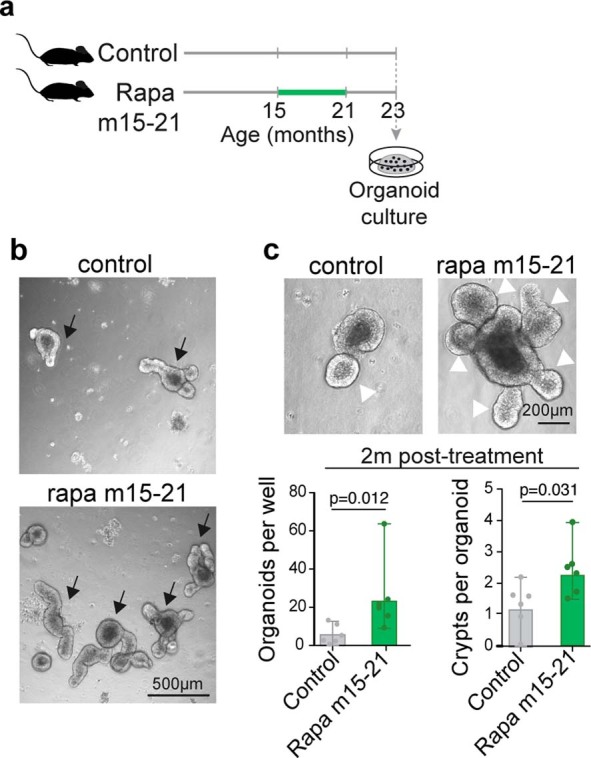
Brief rapamycin treatment improves regenerative capacity of the mammalian intestinal epithelium. **a**, Experimental design. **b-c**, Rapamycin increased organoid forming potential of intestinal crypts (arrows) and (j), formation of de novo crypts (white arrowheads), which remained increased 2 months post-treatment (n = 6 mice). Data are mean ± s.d. Two-sided student t-test.

Paneth cell health is critical to the homeostasis of the small intestine, including promoting ISC proliferation and maintenance, which eventually mediates regenerative capacity of the intestinal epithelium^35,36^. We measured the regenerative ability by assessing mouse intestinal epithelial crypts to form clonogenic organoids in vitro. Mice were treated with rapamycin starting from an older age of 15–21 months, followed by a switch to control food for another 2 months (Extended Data Fig. 10a). Interestingly, compared to untreated controls, short-term treatment in older mice increased the organoid-forming potential of intestinal crypts isolated 2 months after drug withdrawal (Extended Data Fig. 10b). Regenerative growth of de novo crypts was also increased in organoids generated from intestines from short-term rapamycin-treated mice (Extended Data Fig. 10c).

Together, these data show that short-term rapamycin exposure in adult mice combated age-related decline in intestinal TJ structure, Paneth cell architecture and gut barrier function, and that these geroprotective effects were equivalent to those seen with chronic drug exposure and lasted long after rapamycin treatment was withdrawn. In addition, our data indicate that brief rapamycin may improve the regenerative capacity of the intestinal epithelium in the long term.

## Discussion

Our study has uncovered a long-lasting effect of short-term rapamycin administration, including prolonged activation of autophagy, reduced age-related gut pathologies and extension of lifespan in *Drosophila*. Brief rapamycin administration in early adult life induced these benefits to the same degree as lifelong treatment, with a key role of the enterocytes in the intestine. The long-term elevation in autophagy was mediated by the lasting increase in LManV and BCAT expression. Importantly, some of these benefits from early, brief rapamycin treatment were also observed in the small intestine of mice, suggesting the ‘rapamycin memory’ is at least partially conserved in this mammalian model. These findings are intriguing in light of the key role of autophagy in an array of age-related diseases, including cancer^37^, immune system dysfunction^8^ and neurodegenerative diseases^38^. Our findings suggest that the geroprotective effects of rapamycin can be achieved by early, short-term treatment, without the adverse effects sometimes seen with chronic, long-term dosing. While our data shed light on a new path to achieve geroprotection via pharmacological interventions, it will be important to determine the temporal clinical dosing regimen that maximizes protection while minimizing side effects.

Our study is not without limitations. Aging phenotypes are often collected from very old mice (>18 months). However, aging phenotypes already appear in middle-aged mice^39,40^ and, indeed, evolutionary analysis indicates that aging is expected to commence with the onset of reproduction and adulthood^41^. In this study, we first assessed at what ages age-related gut phenotypes appear. Having found that they appear already at middle age (12 months old), we investigated the effects of short-term rapamycin treatment in early adulthood on middle-aged mice. Since these phenotypes are further exacerbated at older ages, it will be important to test in future the extent of protection that earlier-life, short-term rapamycin treatment confers in very old mice. Aging research is often limited by the need for long-term experiments, and the findings from our and other laboratories that age-related phenotypes appear and can be studied already at middle age, are of general utility for the field. Any importance of BCAT as a potential mediator of ‘rapamycin memory’ for gut dysplasia and lifespan in *Drosophila* should be assessed with a larger sample. A further challenge in this study was the measurement of autophagy in mice. Standard techniques to measure autophagy in mice, such as enumerating p62 punctae, showed no effect of either chronic or short-term rapamycin treatment. Intestinal cells may compensate for long-term drug treatment to restore normal levels of autophagy, and may also be particularly responsive to nutrient intake, which greatly affects autophagy. Food intake of the mice was not controlled and nor were they fasted overnight before tissue collection; therefore, the variability of timing of food consumption in different mice may have masked any effect of rapamycin on autophagy. Intestinal organoids were assessed in mice briefly treated with rapamycin at a later age (15–21 months) than in other experiments, and a chronic rapamycin group was not included due to a limited number of old mice available, so more detailed study is warranted. We also limited our study to female flies and mice. This is justified in flies as males do not show increased lifespan in response to rapamycin treatment^2,15^, but in mice there are sex differences in the responses of lifespan and age-related pathologies to rapamycin treatment^11,42^. In future, it will be of great interest to see if short-term rapamycin treatment in early adulthood can delay aging of other organ systems, such as cardiovascular, immune and cognitive function, and increase the survival of mice to the same degree as chronic rapamycin treatment in both sexes.

## Methods

### Fly husbandry and strains

The white Dahomey (*w*^*Dah*^), *Wolbachia*-positive females were used, unless otherwise stated. Fly stocks were maintained at 25 °C on a 12 h light/dark cycle, at constant humidity (60%), and reared on sugar/yeast/agar (SYA) diet, at standard larval density, by collecting eggs on grape juice plates, washing with PBS and pipetting 20 μl of the eggs into each culture bottle. Eclosing adult flies were collected over 18 h and mated for 48 h, then sorted into single sexes. Female flies were used. All mutants and transgenes were backcrossed for at least six generations into the *w*^*Dah*^ background, except the *UAS-BCAT-RNAi* line. The following strains were used in the study: *T*iGS^43^, 5966GS^44^, 5961GS^16,45^, Resille-GFP from the Flytrap project^46^, *UAS*-*Atg5*-RNAi and *UAS*-*Atg1* OE (GS10797) obtained from the Kyoto Drosophila Genetic Resource Center^47,48^, UAS-*LManV*^49^, *UAS-LManV* RNAi (GD13040) obtained from Vienna Drosophila Stock Center, *UAS-BCAT* RNAi (38363) obtained from Bloomington Drosophila Stock Center and *UAS-H3/H4* generated in this laboratory^22^.

### Standard media and rapamycin treatment for *Drosophila*

Standard SYA medium was used, containing per liter (l) 100 g autolyzed yeast powder (brewer’s yeast, MP Biomedicals), 50 g sucrose (Sigma), 15 g agar (Sigma), 3 ml propionic acid (Sigma), 30 ml Nipagin (methyl 4-hydroxybenzoate) and distilled water to 1 l. SYA diet was prepared as described before^50^. Rapamycin was dissolved in ethanol, and added to the food in concentration of 200 μM.

### Lifespan assays

Females were placed into vials containing experimental diets and drugs, at a density of 20 flies per vial, and transferred into vials containing fresh food every 2–3 d, when the number of dead flies was scored. Sample size and analyses of all lifespan data are shown in Supplementary Tables 1–9.

### Mouse husbandry and rapamycin treatment

Female C3B6F1 hybrids were used and were bred in an in-house animal facility at the Max Planck Institute for Biology of Ageing. C3B6F1 hybrids were generated by a cross between C3H female and C57BL/6J male mice, obtained from Charles River Laboratories. Four-week-old mice were housed in individually ventilated cages, in groups of five mice per cage, under specific-pathogen-free conditions at 21 °C, with 50–60% humidity and a 12-h light/dark cycle. Mice had ad libitum access to chow (Ssniff Spezialdiäten; 9% fat, 24% protein, 67% carbohydrates) and drinking water at all times. Mouse experiments were performed in accordance with the recommendations and guidelines of the Federation of the European Laboratory Animal Science Association (FELASA), with all protocols approved by the Landesamt für Natur, Umwelt und Verbraucherschutz, Nordrhein-Westfalen, Germany (reference nos. 84-02.04.2017.A074 and 84-02.04.2015.A437). For 6-month post-switch measurements, rapamycin was added at concentration of 14 ppm (mg of drug per kg of food), encapsulated in Eudragit S100 (Evonik). Control chow contained Eudragit encapsulation medium only. Rapamycin treatment was initiated at 3 months of age and was administered either continuously until 12 months of age (rapamycin chronic group) or until month 6, after which the switch-off group received control chow for an additional 6 months (rapamycin 3–6-month group). All mice from the 6-month post-switch experiment were killed at 12 months of age. For the 2-month post-switch organoid experiment, rapamycin treatment (42 ppm, 1-week-on/1-week-off intervals) was started at 15 months of age and terminated at 21 months of age, after which the switch-off group received control chow for an additional 2 months. Mice were killed at 23 months of age and 2 months after treatment.

### Western blot analysis

Tissues were lysed in 2× Laemmli buffer (head, thorax and fat body) and proteins denatured at 95 °C for 5 min. Proteins from gut were extracted using 20% trichloric acid, washed in 1 M Tris buffer (no pH change), resuspended in 2× Laemmli buffer and denatured at 95 °C for 5 min. Proteins (10 μg) were separated using pre-stained SDS–PAGE gels (Bio-Rad) and wet-transferred onto a 0.45-μm nitrocellulose membrane (GE Healthcare). Blots were incubated with primary p-T389-S6K (CST, 9209), S6K2, Atg8 and Ref-2-P^51^ antibodies (all diluted in a 1:1,000 ratio). Horseradish peroxidase-linked secondary antibodies, Goat Anti-Rabbit IgG Antibody (Sigma, 12-348; 1:10,000 dilution) or Goat Anti-Mouse IgG Antibody (Sigma, 12-349; 1:10,000 dilution) were used. Signal was developed using ECL Select Western Blotting Detection Reagent (GE Healthcare). Images were captured using a ChemiDoc XRS^+^ System with Image Lab (v5.1, Bio-Rad) and band intensity was analyzed using Fiji (v2.1.0).

### Immunostaining of fly intestines

Flies were immobilized on ice and guts were dissected in ice-cold PBS. Dissected guts were immediately fixed in 4% formaldehyde for 30 min, washed in 0.2% Triton-X/PBS (PBST) and blocked in 5% BSA/PBS for 1 h on a shaker. Gut tissues were incubated with primary pH3 (CST, 9701; 1:500 dilution), dpErk (CST, 4370; 1:400 dilution) or lysozyme (Thermo Fisher Scientific, PA5-16668; 1:100 dilution) solutions in 5% BSA overnight at 4 °C, followed by incubation in secondary Alexa Fluor 594 donkey anti-rabbit antibody (Thermo Fisher Scientific, A21207; 1:1,000 dilution). Guts were mounted in mounting medium containing DAPI (Vectashield, H1200), scored and imaged using a Leica inverted microscope for the cell division assay and confocal SP8-DLS for the dpErk staining, with Leica Application Suite X software (v3.x, Leica Microsystems). pH3 and dpErk imaging was performed on the R2 region proximal to the proventriculus and for each intestine three adjacent images were taken.

### Gut turnover assay

*w*^*Dah*^ flies were crossed to the *esg*^*ts*^ F/O flies (w; *esg-Gal4, tubGal80*^*ts*^*, UAS-GFP; UAS-flp, Act* > *CD2* > *Gal4*). Crosses were maintained and progeny were raised at 18 °C. Following a 3-d mating at 18 °C, female flies were distributed into vials containing ethanol or rapamycin and kept at 18 °C for 15 d. On day 15, a subgroup of flies was switched from rapamycin to ethanol food and all experimental groups were transferred to 29 °C. Flies were maintained at 29 °C for 10 and 20 d, after which guts were dissected, fixed in 4% formaldehyde, and mounted in DAPI-containing mounting medium (Vectashield, H1200). Samples were imaged under a confocal microscope (Leica TCS SP8-X), and images analyzed using ImageJ. The GFP-marked regions represent ISCs and their newly generated progenitor cells, and the GFP-marked area compared to the total corresponding gut area indicates the gut turnover rate. Images were obtained from R4 and R5 intestinal regions.

### Gut barrier analysis

Flies were aged for 65 d on standard SYA diet then transferred into vials containing SYA food with 2.5% (wt/vol) FD&C blue dye no. 1 (Fastcolors). The proportion of blue (whole body is blue) or partially blue (at least two-thirds of body is blue) flies was scored 24 h after exposure to the blue food.

### Imaging of gut dysplasia

Guts were dissected in ice-cold PBS, fixed in 4% formaldehyde for 30 min and mounted in DAPI-containing mounting medium (Vectashield, H1200). Endogenous GFP and DAPI were imaged using a confocal microscope. For each condition, 6–14 guts were imaged. The area affected by tumors was measured using the measure function in Fiji software (v2.1.0), and the average proportion of the affected area for each gut was calculated.

### Cyto-ID and LysoTracker staining, imaging and image analysis

Flies were immobilized on ice, dissected in PBS and stained with Cyto-ID (Enzo Life Sciences; 1:1,000 dilution) for 30 min, then stained with LysoTracker Red DND-99 (Thermo Fisher Scientific; 1:2,000 dilution) and Hoechst 33342 (Sigma, 1 mg ml^−1^; 1:1,000 dilution) for 3 min in 12-well plates on a shaker. Immediately after staining, guts were mounted (Vectashield, H1000) and imaged using a Leica SP8-X confocal microscope. For each gut preparation, an area proximal to the proventriculus was imaged to control for variation across different gut regions, and three adjacent images per gut were captured. Images were analyzed using IMARIS software (v8.2, Oxford Instruments). This experiment was carried out under blinded conditions.

### Lipopolysaccharide-binding protein measurement in mouse plasma

LBP was measured in mouse plasma samples by ELISA assay according to the manufacturer’s instructions (HyCult Biotech, HK: 205).

### Transmission electron microscopy

The intestine was fixed in 2% glutaraldehyde/2% formaldehyde in 0.1 M cacodylate buffer (pH 7.3) for 48 h at 4 °C. Afterwards, samples were rinsed in 0.1 M cacodylate buffer (AppliChem) and post-fixed with 2% osmium tetroxid (Science Services) in 0.1 M cacodylate buffer for 2 h at 4 °C. Samples were dehydrated through an ascending ethanol series (AppliChem) and embedded in epoxy resin (Sigma-Aldrich). Ultrathin sections (70 nm) were cut with a diamond knife (Diatome) on an ultramicrotome (EM-UC6, Leica Microsystems) and placed on 100-mesh copper grids (Science Services). The sections were contrasted with 1.5% uranyl acetate (Plano) and lead citrate (Sigma-Aldrich). Images were acquired with a transmission electron microscope (JEM 2100 Plus, JEOL) and a OneView 4K camera (Gatan) with DigitalMicrograph software (v3.x, Gatan) at 80 kV at room temperature (RT). For each mouse and for each measured phenotype, ten random images were taken and the final score for each mouse was calculated as a mean value obtained from ten images. Imaging and scoring of electron microscope data were carried out under blinded conditions.

### Isolation of mouse intestinal crypts and organoid culture

Mouse jejunal sections were used to isolate crypts according to the manufacturer’s instructions (STEMCELL Technologies, 28223). Complete IntestiCult medium was exchanged every 2–3 d and organoid numbers and de novo crypts were scored on days 5 and 7. This experiment was carried out under blinded conditions.

### Immunostaining of mouse tissues

Jejunal sections were fixed in 4% paraformaldehyde, embedded in paraffin and sectioned. Slides were deparaffinized and antigen retrieval was performed by boiling with pH 6 citrate buffer. Primary antibodies used were: p62/SQSTM (Abcam, 56416; 1:100 dilution), pH3 (CST, 4370; 1:100 dilution), lysozyme (Thermo Fisher Scientific, PA5-16668; 1:300 dilution) and Man2B1 (St John’s Laboratory, 640-850; 1:100 dilution). Primary antibodies were detected using Alexa Flour 488-, Alexa Flour 594- and Alexa Flour 633-conjugated anti-rabbit or anti-mouse secondary antibodies (Thermo Fisher Scientific; 1:500 dilution). Sections were mounted in DAPI-containing mounting medium (Vectashield H1200) and imaged using a confocal Leica SP8-DLS or SP8-X microscope, with Leica Application Suite X software (v3.x, Leica Microsystems).

### RNA isolation and RT–qPCR

Fly guts were dissected and frozen on dry ice, and were stored at −80 °C. Total RNA from guts of 15 females was extracted using TRIzol (Invitrogen) according to the manufacturer’s instructions. cDNA was generated by using total RNA with random hexamers and the SuperScript III First Strand system (Invitrogen). RT–qPCR was performed using LManV-specific TaqMan probes and primers (Thermo Fisher, Dm01809748_gH) on a QuantStudio 6 instrument with QuantStudio Real-Time PCR software v1.1 (Thermo Fisher Scientific) by following the manufacturer’s instructions.

### Peptide generation and tandem mass tag labeling

In total, 20 μl of lysis buffer (6 M guanidine chloride, 2.5 mM TCEP,10 mM 2-chloroacetamide and 100 mM Tris-HCl) was added to 25 guts, and tissues were homogenized using a hand homogenizer. Homogenates were heated at 95 °C for 10 min and subsequently sonicated using the Bioruptor (10 cycles, 30 s sonication/30 s break, high performance). Samples were centrifuged for 20 min at 2,000*g* and supernatant was diluted tenfold in 20 mM Tris. Protein concentration in the supernatant was measured using a NanoDrop and a 1:200 dilution (wt/wt) of trypsin (Promega, mass spectrometry grade) was added to 200 µg of sample. Trypsin digestion was performed overnight at 37 °C and stopped by the addition of 50% of formic acid (FA) to a final concentration of 1%. Peptide cleanup was carried out using an OASIS HLB Plate. Wetting of the wells was performed by the addition of 200 µl of 60% acetonitrile/0.1% FA and equilibration adding 400 µl of 0.1% FA. The sample and 100 µl of 0.1% FA were loaded into the wells and peptides eluted by the addition of 80 µl of 60% acetonitrile/0.1% FA. Peptides were air-dried using the SpeedVac and the pellet resuspended in 60 µl of 0.1% FA. Then, 15 µg of peptides was dried in SpeedVac and used for tandem mass tag (TMT) labeling. The pellet was dissolved in 17 µl of 100 mM triethylammonium bicarbonate and 41 µl of anhydrous acetonitrile was added. Samples were incubated for 10 min at RT with occasional vortexing, followed by the addition of 8 µl of TMT label and subsequent incubation for 1 h at RT. The labeling reaction was stopped by the addition of 8 µl of 5% hydroxylamine and incubation for 15 min. Samples were air-dried in the SpeedVac, resuspended in 50 µl of 0.1% FA and cleaned with an OASIS HLB Plate as previously described. Four replicates per condition and 25 intestines per replicate were used for peptide generation and TMT labeling for proteomics analysis.

### High-pH fractionation

Pooled TMT-labeled peptides were separated on a 150-mm, 300-μm OD, 2-μm C18, Acclaim PepMap (Thermo Fisher Scientific) column using an Ultimate 3000 (Thermo Fisher Scientific). The column was maintained at 30 °C. Buffer A was 5% acetonitrile 0.01 M ammonium bicarbonate, while buffer B was 80% acetonitrile 0.01 M ammonium bicarbonate. Separation was performed using a segmented gradient from 1% to 50% buffer B, for 85 min and 50% to 95% for 20 min with a flow of 4 μl. Fractions were collected every 150 s and combined into nine fractions by pooling every ninth fraction. Pooled fractions were dried in a Concentrator plus (Eppendorf), resuspended in 5 μl 0.1% FA from which 2 μl was analyzed by liquid chromatography with tandem mass spectrometry (LC–MS/MS).

### Liquid chromatography–tandem mass spectrometry analysis

Peptides from each of the nine high-pH fractions were separated on a 25-cm, 75-μm internal diameter PicoFrit analytical column (New Objective) packed with 1.9-μm ReproSil-Pur 120 C18-AQ media (Dr. Maisch) using an EASY-nLC 1200 (Thermo Fisher Scientific). The column was maintained at 50 °C. Buffer A and B were 0.1% FA in water and 0.1% FA in 80% acetonitrile. Peptides were separated on a segmented gradient from 6% to 31% buffer B for 120 min and from 31% to 50% buffer B for 10 min at 200 nl min^−1^. Eluting peptides were analyzed on an Orbitrap Fusion mass spectrometer (Thermo Fisher Scientific) in TMT-SPS mode. Peptide precursor *m/z* measurements were carried out at a resolution of 60,000 in the 350 to 1,500 m/z range with an automatic gain control (AGC) target of 1e6. Precursors with a charge state from 2 to 7 only were selected for collision-induced dissociation fragmentation using 35% collision energy and an isolation window width of 0.7. The *m/z* values of the peptide fragments, MS/MS, were measured in the IonTrap at a ‘rapid’ scan rate, a minimum AGC target of 1e4 and a 100-ms maximum injection time. Upon fragmentation, precursors were put on a dynamic exclusion list for 45 s. The top ten most intense MS/MS peaks were subjected to multi-notch isolation with an AGC target of 5e4 and a 86-ms maximum injection time and further fragmented using higher energy collision dissociation with 65% collision energy. The *m/z* values of the fragments, MS3, were measured in the Orbitrap at a resolution of 50,000. The cycle time was set to 2 s.

### Protein identification and quantification

The raw data were analyzed with MaxQuant (version 1.5.2.8)^52^ using the integrated Andromeda search engine^53^. Peptide fragmentation spectra were searched against the canonical and isoform sequences of the *Drosophila melanogaster* reference proteome (proteome ID UP000000803, downloaded September 2018 from UniProt). Methionine oxidation and protein N-terminal acetylation were set as variable modifications; cysteine carbamidomethylation was set as fixed modification. The digestion parameters were set to ‘specific’ and ‘Trypsin/P,’ The minimum number of peptides and razor peptides for protein identification was 1; the minimum number of unique peptides was 0. Protein identification was performed at peptide spectrum matches and protein false discovery rate of 0.01. The ‘second peptide’ option was on. The quantification type was set to ‘Reporter ion MS3’ and ‘10-plex TMT’. Before the analysis, the TMT correction factors were updated based on the values provided by the manufacturer.

#### Bioinformatics

##### Proteomics data analysis

Intensity values were log_2_ transformed and each sample was separately *z*-transformed. For simpler interpretation, the *z*-scores were rescaled to approximately their original scale by multiplying each *z*-score with the overall standard deviation of the original log_2_ transformed data and adding back the overall mean of the original log_2_ transformed data. The normalized data were filtered for proteins that were detected in at least three replicates per biological group and proteins annotated as contaminant or reverse identification were removed. Missing values after filtering were imputed using the impute.knn function from the impute package (version 1.56.0)^54^. Differential expression analysis was performed using the limma package (version 3.38.3)^55^. *P* values were corrected for multiple testing using the Benjamini–Hochberg procedure and a significance threshold of 0.05 was used to determine significant differential expression. Differential expression was determined between the following biological groups: 25-day-old flies chronically treated with rapamycin versus 25-day-old control flies and 25-day-old flies treated with rapamycin from days 1–15 versus 25-day-old control flies. The normalized data after batch effect removal with the removeBatchEffect function from the limma package were used for principal-component analysis using the prcomp function from Rstudio (R package version 3.5.3).

##### Gene Ontology term enrichment

The topGO package (version 2.32.0)^56^ with the annotation package org.dm.for exampledb^57^ was used for GO term enrichment analysis. The weight01 Fisher procedure^58^ was used with a minimal node size of five. The enrichment of each term was defined as the log2 of the number of significant genes divided by the number of expected genes. Protein groups of interest were tested for enrichment against a universe of all detected proteins. Only significantly enriched terms with a minimum of three significant proteins and a maximum of 300 annotated genes were used in the cell plot.

### Statistics and reproducibility

No statistical methods were used to predetermine sample sizes but our sample sizes are similar to those reported in previous publications^2,22,46^. No specific methods were used to randomly allocate samples to groups. Experiments were carried out in an unblinded fashion unless otherwise stated. No data were excluded from the analysis. Statistical analysis was performed in Prism (v7.0, GraphPad) except for survival analysis, and data distribution was assumed to be normal but this was not formally tested. Statistical tests for each experiment are mentioned in the corresponding figure legends. Survival data were analyzed with a log-rank test and CPH analysis, using Excel 2016 (Microsoft) and Jmp (v10, SAS Institute) software, respectively. Bioinformatics analysis was performed using Rstudio (R version 3.5.3).

### Reporting summary

Further information on research design is available in the Nature Research Reporting Summary linked to this article.

## Supplementary information

Supplementary Information Supplementary Tables 1–9

Reporting Summary

## Data Availability

The mass spectrometry proteomics data have been deposited to the ProteomeXchange Consortium via the PRIDE partner repository with the dataset identifier PXD020820. Source data are provided with this paper. All other data supporting the findings of this study are available from the corresponding author upon reasonable request.

## Source data

Source Data Fig. 1 Unprocessed western blot for Fig. 3b,c.
Source Data Fig. 2 Unprocessed western blot for Fig. 5a.
Source Data Fig. 3 Unprocessed western blot for Extended Data Fig. 2.
Source Data Fig. 4 Unprocessed western blot for Extended Data Fig. 4a.
