# Supplementary material for: Long-lasting geroprotection from brief rapamycin treatment in early adulthood by persistently increased intestinal autophagy
Source: Nat Aging. 2022 Aug 29;2(9):824–36. doi: 10.1038/s43587-022-00278-w (PMC10154223; doi:10.1038/s43587-022-00278-w)
Supplement: Supplementary file 1 — Supplementary Information Supplementary Tables 1–9 [file 43587_2022_278_MOESM1_ESM.pdf]

nature portfolio

https://doi.org/10.1038/s43587-022-00278-w

Supplementary information

Long-lasting geroprotection from brief rapamycin treatment in early adulthood by persistently increased intestinal autophagy

In the format provided by the authors and unedited

Supplementary Information

Supplementary Table 1. Statistical analysis of survival data presented in Fig. 1a.

30-day switch – data before day 30 were censored
n=400/treatment

p-value (log rank)

	control	rapamycin	rapamycin 30-end	rapamycin 1-30
median (d)	72.5	82	78	82
mean (d)	73.2	82.3	77.6	81.3
control		6.84E-27	2.13E-06	2.39E-13
rapamycin chronic			1.04E-13	0.087215
rapamycin 30-end				2.88E-06

Cox Proportional Hazard (CPH) analysis
Risk Ratios

Level 1	/Level 2	Risk Ratio	p	Lower 95%	Upper 95%
rapamycin	control	0.4309059	<0.0001	0.3734110	0.4977745
rapamycin 1-30	rapamycin chronic	1.1543417	0.1266	0.9601738	1.3877745
rapamycin 30-end	rapamycin chronic	1.4612031	<0.0001	1.2173019	1.7539727
rapamycin 1-30	rapamycin 30-end	0.6843676	<0.0001	0.5701343	0.8214889

Supplementary Table 2. Statistical analysis of survival data presented in Fig. 1b,c.

n=400/treatment

Rapamycin day 45-end – data before day 45 were censored
p-value (log rank)

	control	rapamycin	rapamycin 45-end
median (d)	75.5	82	78
mean (d)	73.8	83.2	76.3
control		1.22E-28	0.00030
rapamycin chronic			3.7E-12

Rapamycin day 60-end – data before day 60 were censored
p-value (log rank)

	control	rapamycin	rapamycin 60-end
median (d)	75.5	84	75.5
mean (d)	76.9	84	77.7
control		1.09E-21	0.256315
rapamycin chronic			5.38E-16

Cox Proportional Hazard (CPH) analysis
Parameter Estimates

	Term	Estimate	SE	p
d30 v d45 start	rapamycin	0.13680	0.025308	<0.0001
	timing	0.00355	0.024808	0.8861
	rapa*timing	0.00975	0.024808	0.6943
d45 v d60 start	rapamycin	0.07538	0.027004	0.0052
	timing	0.05899	0.026677	0.0267
	rapa*timing	0.08447	0.026677	0.0016

Supplementary Table 3. Statistical analysis of survival data presented in Fig. 1d,e and 2a.

15-day switch
n=400/treatment

p-value (log rank)

	control	rapamycin	rapamycin 1-30	rapamycin 15-30	rapamycin 1-15
median (d)	72.5	77.5	80	77.5	77.5
mean (d)	72.1	77.7	77.5	76.0	77.5
maximum (d)	89	91.5	91.5	91.5	91.5
control		1.2944E-10	2.0562E-13	7.585E-07	1.2016E-11
rapamycin chronic			0.14736386	0.1908386	0.50923849
rapamycin 1-30				0.0153711	0.51293879
rapamycin 15-30					0.07282181

Cox Proportional Hazard (CPH) analysis
Risk Ratios

Level 1	/Level 2	Risk Ratio	p	Lower 95%	Upper 95%
rapamycin	control	0.6522501	<0.0001	0.565943	0.751711
rapamycin 1-15	rapamycin chronic	0.9632981	0.6074	0.834742	1.111281
rapamycin 15-30	rapamycin chronic	1.0933126	0.2117	0.950452	1.257805
rapamycin 1-15	rapamycin 15-30	0.8809997	0.0792	0.764565	1.014886

Supplementary Table 4. Statistical analysis of survival data presented in Extended Data Fig. 2e,f.

TiGS/UAS-S6K const.act.
n=200/treatment

p-value (log rank)

	EtOH	RU	Rapa	Rapa + RU	RU 1-30	Rapa 1-30	Rapa + RU 1-30
median (d)	67	67	69	71	65	69	69
mean (d)	66.5	65.8	70.4	71.4	63.8	67.3	69.6
maximum (d)	75.5	75.5	82.5	82.5	75.5	80	82.5
EtOH		0.37293	9.9E-05	9.27E-06	0.424	0.029619	0.001376
RU			0.00438	0.002346	0.8139	0.396167	0.071006
Rapamycin				0.863832	0.0017	0.016441	0.16678
Rapamycin + RU					0.0013	0.011603	0.229435
RU 1-30						0.225444	0.020668
Rapamycin 1-30							0.23175

Cox Proportional Hazard (CPH) analysis
Risk Ratios

Level 1	/Level 2	Risk Ratio	p	Lower 95%	Upper 95%
rapa chronic	control	0.69202	<0.0001	0.587187	0.8156
induced chronic	non-induced chronic	0.94363	0.4864	0.801264	1.1112
rapa 1-30	control	0.0799	0.0064	0.680384	0.9388
induced 1-30	non-induced 1-30	0.90581	0.2265	0.771479	1.0633

Parameter Estimates

	Term	Estimate	SE	p
chronic	rapamycin	0.18407	0.0418	<0.0001
	induction	-0.0290	0.0416	0.4864
	rapamycin*induction	-0.0271	0.0418	0.5164
day 1-30	rapamycin	0.11219	0.0410	0.0064
	induction	-0.0494	0.0400	0.2265
	rapamycin*induction	-0.0045	0.0408	0.911

Supplementary Table 5. Statistical analysis of survival data presented in Fig. 4a,b.

5966GS/UAS-Atg5 [RNAi]
n=400/treatment

p-value (log rank)

	EtOH	RU	Rapa	Rapa + RU	RU 1-15	Rapa1-15	Rapa+ RU 1-15
median (d)	57	59	61.5	61.5	61.5	64	61.5
mean (d)	58.2	59.1	62.7	59.9	59.1	63.7	60.9
maximum (d)	68	70.5	70.5	68	70.5	70.5	70.5
EtOH		0.467679	1.53E-05	0.156857	0.43617	8.8E-10	0.012342
RU			7.48E-05	0.253446	0.84502	2.4E-08	0.056321
rapamycin				0.000263	9.8E-05	0.03165	0.030291
rapamycin + RU					0.51559	5.4E-09	0.327552
RU 1-15						7.4E-09	0.097373
rapamycin 1-15							8.44E-05
rapamycin + RU 1-15							

Cox Proportional Hazard (CPH) analysis
Risk Ratios

Level 1	/Level 2	Risk Ratio	p	Lower 95%	Upper 95%
rapa chronic	control	0.740356	0.0006	0.624593	0.87815
induced chronic	non-induced chronic	1.123287	0.1774	0.948662	1.33023
rapa 1-15	control	0.553101	<0.0001	0.461037	0.66278
induced 1-15	non-induced 1-15	0.960025	0.6559	0.802404	1.149

Parameter Estimates

	Term	Estimate	SE	p
chronic	rapamycin	0.150312	0.043435	0.0006
	induction	0.05813	0.043093	0.11774
	rapamycin*induction	-0.083593	0.043034	0.0516
day 1-15	rapamycin	0.203859	0.044964	<0.0001
	induction	0.0543	0.044685	0.2246
	rapamycin*induction	-0.074401	0.044552	0.0948

Supplementary Table 6. Statistical analysis of survival data presented in Fig. 4c,d.

5961GS/UAS-Atg5 ^{RNAi}
n=200/treatment

p-value (log rank)

	EtOH	RU	Rapa	Rapa + RU	RU 1-15	Rapa 1-15	Rapa + RU 1-15
median (d)	54.5	54.5	61.5	61.5	54.5	61.5	61.5
mean (d)	54.1	55.4	59.7	60.3	55.3	62.2	62.4
maximum (d)	68	70.5	69	68	61.5	70.5	70.5
EtOH		0.233667	3.4E-06	3.49E-06	0.5718	8.2E-13	1.93E-12
RU			0.001097	0.001491	0.4706	2.8E-09	1.1E-08
rapamycin				0.73857	1E-05	0.00529	0.011967
rapamycin + RU					2.4E-05	0.00053	0.001288
RU 1-15						8.2E-12	5.4E-12
rapamycin 1-15							0.386465
rapamycin + RU 1-15							

Cox Proportional Hazard (CPH) analysis
Risk Ratios

Level 1	/Level 2	Risk Ratio	p	Lower 95%	Upper 95%
rapa chronic	control	0.630867	<0.0001	0.526928	0.75437
induced chronic	non-induced chronic	0.929143	0.4213	0.776204	1.11124
rapa 1-15	control	0.553101	<0.0001	0.461037	0.66278
induced 1-15	non-induced 1-15	0.960025	0.6559	0.802404	1.149

Parameter Estimates

	Term	Estimate	SE	p
	rapamycin	0.23033	0.045737	<0.0001
chronic	induction	-0.036746	0.045736	0.4213
	rapamycin*induction	-0.022517	0.045743	0.6225
	rapamycin	0.138892	0.03734	0.0002
day 1-15	induction	0.15442	0.037407	<0.0001
	rapamycin*induction	-0.024520	0.03722	0.5101

Supplementary Table 7. Statistical analysis of survival data presented in Fig. 5b,c.

5966GS/UAS-Atg1 ^{OE}
n=200/treatment (Fig. 5b) and 160/treatment (Fig. 5c)

p-value (log rank)

	EtOH	RU	Rapa	Rapa+ RU
median (d)	60	67	64.5	64.5
mean (d)	59.2	66.4	64.4	63.5
maximum (d)	71.5	71.5	71.5	71.5
EtOH		3.6E-11	5.68E-06	8.49E-05
RU			0.046318	0.004648
rapamycin				0.506055
rapamycin + RU				

	EtOH	RU 1-15	Rapa 1-15	Rapa + RU 1-15
median (d)	60.5	67.5	67.5	67.5
mean (d)	60.3	66.8	67.3	67.8
maximum (d)	74.5	74.5	74.5	74.5
EtOH		6.72E-5	1.00E-06	1.84E-07
RU 1-15			0.371120	0.212741
rapamycin 1-15				0.697781
rapamycin + RU 1-15				

Cox Proportional Hazard (CPH) analysis
Risk Ratios

Level 1	/Level 2	Risk Ratio	p	Lower 95%	Upper 95%
rapa chronic	control	0.567652	<0.0001	0.461557	0.70218
induced chronic	non-induced chronic	0.380319	<0.0001	0.307387	0.47305
rapa 1-15	control	0.725947	0.0002	0.613941	0.85838
induced 1-15	non-induced 1-15	0.790212	0.0059	0.668278	0.93439

Parameter Estimates

	Term	Estimate	SE	p
	rapamycin	-0.283123	0.053447	<0.0001
chronic	induction	-0.483371	0.054918	<0.0001
	rapamycin*induction	0.156973	0.052663	0.0036
	rapamycin	0.160138	0.042750	0.0002
day 1-15	induction	-0.117726	0.042755	0.0059
	rapamycin*induction	-0.107443	0.042741	0.0119

Supplementary Table 8. Statistical analysis of survival data presented in Extended Data Fig. 4c.

5966GS/UAS-H3/H4 ^{OE}
n=200/treatment

p-value (log rank)

	EtOH	RU	RU 1-15
median (d)	66.6	71.0	66.6
mean (d)	66.9	71.0	67.5
maximum (d)	82.0	89.0	86.0
control		7.38E-06	0.59709
RU			0.00011

Supplementary Table 9. Statistical analysis of survival data presented in Extended Data Fig. 7c.

5966GS/UAS-BCAT ^{RNAi}
n=180/treatment

p-value (log rank)

	EtOH	RU 1-15	Rapa 1-15	Rapa + RU 1-15
median (d)	79.0	79.0	81.5	79.0
mean (d)	80.6	80.5	82.2	80.9
maximum (d)	86.0	88.5	91.0	88.5
EtOH		0.699135	7.83E-04	0.050575
RU 1-15			0.002971	0.138767
rapamycin 1-15				0.150979
rapamycin + RU 1-15				
