# Supplementary material for: Long-lasting geroprotection from brief rapamycin treatment in early adulthood by persistently increased intestinal autophagy
Source: Nat Aging. 2022 Aug 29;2(9):824–36. doi: 10.1038/s43587-022-00278-w (PMC10154223; doi:10.1038/s43587-022-00278-w)
Supplement: Supplementary file 2 — Reporting Summary [file 43587_2022_278_MOESM2_ESM.pdf]

nature portfolio

Corresponding author(s): Linda Partridge
Last updated by author(s): Jul 29, 2022

Reporting Summary

Nature Portfolio wishes to improve the reproducibility of the work that we publish. This form provides structure for consistency and transparency in reporting. For further information on Nature Portfolio policies, see our Editorial Policies and the Editorial Policy Checklist.

Statistics

For all statistical analyses, confirm that the following items are present in the figure legend, table legend, main text, or Methods section.

n/a	Confirmed	
☐	☒	The exact sample size (*n*) for each experimental group/condition, given as a discrete number and unit of measurement
☐	☒	A statement on whether measurements were taken from distinct samples or whether the same sample was measured repeatedly
☐	☒	The statistical test(s) used AND whether they are one- or two-sided<br>*Only common tests should be described solely by name; describe more complex techniques in the Methods section.*
☐	☒	A description of all covariates tested
☐	☒	A description of any assumptions or corrections, such as tests of normality and adjustment for multiple comparisons
☐	☒	A full description of the statistical parameters including central tendency (e.g. means) or other basic estimates (e.g. regression coefficient) AND variation (e.g. standard deviation) or associated estimates of uncertainty (e.g. confidence intervals)
☐	☒	For null hypothesis testing, the test statistic (e.g. *F*, *t*, *r*) with confidence intervals, effect sizes, degrees of freedom and *P* value noted<br>*Give P values as exact values whenever suitable.*
☒	☐	For Bayesian analysis, information on the choice of priors and Markov chain Monte Carlo settings
☒	☐	For hierarchical and complex designs, identification of the appropriate level for tests and full reporting of outcomes
☐	☒	Estimates of effect sizes (e.g. Cohen's *d*, Pearson's *r*), indicating how they were calculated

Our web collection on statistics for biologists contains articles on many of the points above.

Software and code

Policy information about availability of computer code

Data collection	Immunofluorescence image: Leica Application Suite X software v3.x, Leica microsystems<br>Western blot: ChemiDoc™ XRS+ System + Image Lab software v5.1, Biorad<br>Electron microscopy image acquistion: DigitalMicrograph software v3.x, Gatan<br>qRT-PCR: QuantStudio 6 system + QuantStudio Real-Time PCR software v1.1, Thermo Fisher Scientific<br>Proteomics: Orbitrap Fusion mass spectrometer, Thermo Fisher Scientific
Data analysis	Lysotracker- and Cyto-ID-stained puncta image: IMARIS software v8.2, Oxford Instruments<br>Western blot and Immunofluorescence image: Fiji software v2.1.0<br>Proteomics analysis: RStudio (R v.3.5.3) with publicly-available R packages: limma, topGO (v2.32.0), annotation package org.dm.e.g.db.<br>Statistical analysis except for survival data: Prism v7.0, GraphPad<br>Survival data: Excel 2016,Microsoft and Jmp v10, SAS Institute<br>Proteomic raw data: MaxQuant v1.5.2.8

For manuscripts utilizing custom algorithms or software that are central to the research but not yet described in published literature, software must be made available to editors and reviewers. We strongly encourage code deposition in a community repository (e.g. GitHub). See the Nature Portfolio guidelines for submitting code & software for further information.

Data

Policy information about availability of data

All manuscripts must include a data availability statement. This statement should provide the following information, where applicable:

- Accession codes, unique identifiers, or web links for publicly available datasets
- A description of any restrictions on data availability
- For clinical datasets or third party data, please ensure that the statement adheres to our policy

The mass spectrometry proteomics data have been deposited to the ProteomeXchange Consortium via the PRIDE partner repository with the dataset identifier PXD020820. Complete immunoblot images containing all replicates are available as Source Data files. All other data supporting the findings of this study are available from the corresponding author upon reasonable request.

Field-specific reporting

Please select the one below that is the best fit for your research. If you are not sure, read the appropriate sections before making your selection.

☒ Life sciences ☐ Behavioural & social sciences ☐ Ecological, evolutionary & environmental sciences

For a reference copy of the document with all sections, see nature.com/documents/nr-reporting-summary-flat.pdf

Life sciences study design

All studies must disclose on these points even when the disclosure is negative.

Sample size	Sample size for survival analyses were based on prior, published studies by our lab investigating the effects of rapamycin on lifespan, TORC1 inhibition, autophagy induction and gut pathologies (Bjedov et al, 2010; Castillo-Quan et al., 2019, Lu et al., 2021).<br>To investigate the sample size needed to obtain robust results as to whether rapamycin affects growth of intestinal organoids, previously published studies were used: n=6 (Pentinmikko et al., 2019); n=6 (Mihaylova et al., 2018); n=8 (Nalapareddy et al., 2017); n=3 (Moorefield et al., 2017) mice for intestinal organoid cultures.<br>Sample size for proteomics analysis were based on previously published studies of fly intestine proteome from our lab (Tain et al, 2017).
Data exclusions	No data was excluded.
Replication	All survival experiments were performed 2 or 3 times. All attempts at replication were sucessful. There was no attempt to replicate negative lifespan results, such as Fig. 1b,c,e, Extended Data Fig. 2e-f, and Extended Data Fig. 4c.<br>For each experiment at least 3 biological replicates were used. Mouse electron microscopy data on Paneth cell granules was confirmed by immunostaining with antibody against Paneth-cell-containing enzyme lysozyme.
Randomization	Samples were allocated to treatments/ groups randomly, and steps were taken to reduce batch effects, for example fly lifespans were repeated independently months apart and dissections conducted by more than one person were split across groups, and upon weaning female mice were randomly assigned to cages.
Blinding	Experiments were carried out in an un-blinded fashion unless otherwise stated.

Reporting for specific materials, systems and methods

We require information from authors about some types of materials, experimental systems and methods used in many studies. Here, indicate whether each material, system or method listed is relevant to your study. If you are not sure if a list item applies to your research, read the appropriate section before selecting a response.

Materials & experimental systems

n/a	Involved in the study	
☐	☒	Antibodies
☒	☐	Eukaryotic cell lines
☒	☐	Palaeontology and archaeology
☐	☒	Animals and other organisms
☒	☐	Human research participants
☒	☐	Clinical data
☒	☐	Dual use research of concern

Methods

n/a	Involved in the study	
☒	☐	ChIP-seq
☒	☐	Flow cytometry
☒	☐	MRI-based neuroimaging

Antibodies

Antibodies used	p-T389-S6K (Cell Signalling Technologies, 9209)

Antibodies used	Total S6K, home-made, from this lab (Bjedov et al, 2010)<br>Atg8 and Ref-2-P, home-made, gift from Péter Nagy's lab, Eötvös Loránd University, Budapest, Hungary (Nagy et al, 2015)<br>pH3 (Cell Signalling Technologies, 9701)<br>dpErk (Cell Signalling Technologies, 4370)<br>p62/SQSTM (Abcam, 56416)<br>lysozyme (ThermoFisher Scientific, PA5-16668)<br>Man2B1 (St John's Laboratory, 640-850)<br><br>Goat Anti-Rabbit IgG Antibody, HRP-conjugate (Sigma, 12-348)<br>Goat Anti-Mouse IgG Antibody, HRP-conjugate (Sigma, 12-349)<br>Goat anti-Rabbit IgG (H+L) Cross-Adsorbed Secondary Antibody, Alexa Fluor™ 488 (ThermoFisher Scientific, A-11008)<br>Donkey anti-Rabbit IgG (H+L) Cross-Adsorbed Secondary Antibody, Alexa Fluor™ 594 (ThermoFisher Scientific, A-21207)<br>Goat anti-Mouse IgG (H+L) Cross-Adsorbed Secondary Antibody, Alexa Fluor™ 633 (ThermoFisher Scientific, A-21050)
Validation	p-T389-S6K (Cell Signalling Technologies, 9209) - Validated by the company and the following publication (Wei et al. 2019)<br>S6K – home-made and validated by the following publication (Bjedov et al, 2010)<br>Atg8 and Ref-2-P - home-made and validated by the following publication (Nagy et al, 2015)<br>pH3 (Cell Signalling Technologies, 9701) - validated by the company and the following publication (Dye et al. 2017)<br>dpErk (Cell Signalling Technologies, 4370) - validated by the company and the following publication (Liang et al. 2017)<br>p62/SQSTM (Abcam, 56416) - validated by the company and the following publication (Aragones et al. 2020)<br>lysozyme (ThermoFisher Scientific, PA5-16668) - validated by the company and the following publication (Beyaz et al. 2016)<br>Man2B1 (St John's Laboratory, 640-850) validated by the company (Immunogen: Recombinant fusion protein containing a sequence corresponding to amino acids 640-850 of human MAN2B1 (NP_000519.2))<br><br>HRP-conjugate, Goat anti-Rabbit IgG Antibody and Goat Anti-Mouse IgG Antibody (Sigma) - validated by the company and users<br>Alexa Flour 488-, Alexa Flour 594- and Alexa Flour 633- conjugated anti-rabbit or anti-mouse secondary antibodies (Thermo Fisher Scientific) - validated by the company and users

Animals and other organisms

Policy information about studies involving animals; ARRIVE guidelines recommended for reporting animal research

Laboratory animals	Mus musculus: Female C3B6F1 hybrids were used and were bred in an in-house animal facility at the Max Planck Institute for Biology of Ageing. C3B6F1 hybrids were generated by a cross between C3H female and C57BL/6J male mice, obtained from Charles River Laboratories. Four-week-old mice were housed in individually ventilated cages, in groups of five mice per cage, under specific-pathogen-free conditions at 21°C, with 50-60% humidity and 12h light/dark cycle. Mice had ad libitum access to chow (Ssniff Spezialdiäten GmbH; 9% fat, 24% protein, 67% carbohydrates) and drinking water at all times.<br><br>Drosophila melanogaster: white Dahomey (wDah), Wolbachia positive females was used, unless otherwise stated. Flies were maintained at 25°C on a 12 h light/dark cycle, at constant humidity (60%), and reared on sugar/yeast/agar (SYA) diet. Fly strains used were: TiGS (Slack et al., 2015), 5966GS (Guo et al., 2014), 5961GS (Biteau et al., 2010), P{PTT-un1}CG8668117-2 (Resille-GFP) from the Flytrap project (Regan et al., 2016), UAS-Atg5-RNAi and UAS-Atg1 OE (GS10797), obtained from the Kyoto Drosophila Genetic Resource Center (Ren et al., 2009, Scott et al, 2016), UAS-LManV, UAS-LManV RNAi (GD13040) obtained from Vienna Drosophila Stock Center, UAS-BCAT RNAi (38363) obtained from Bloomington Drosophila Stock Center, UAS-H3/H4 OE generated in this lab (Lu et al. 2021).<br><br>All all relevant details (species, sex, age) were also described in relevant Figure legends.
Wild animals	Our study did not involve any wild animals.
Field-collected samples	Our study did not involve any field-collected samples.
Ethics oversight	Mouse experiments were performed in accordance with the recommendations and guidelines of the Federation of the European Laboratory Animal Science Association (FELASA), with all protocols approved by the Landesamt für Natur, Umwelt und Verbraucherschutz, Nordrhein-Westfalen, Germany (reference numbers: 84-02.04.2017.A074 and 84-02.04.2015.A437).

Note that full information on the approval of the study protocol must also be provided in the manuscript.
