# Supplementary figures and images for: Long-lasting geroprotection from brief rapamycin treatment in early adulthood by persistently increased intestinal autophagy
Source: Nat Aging. 2022 Aug 29;2(9):824–36. doi: 10.1038/s43587-022-00278-w (PMC10154223; doi:10.1038/s43587-022-00278-w)

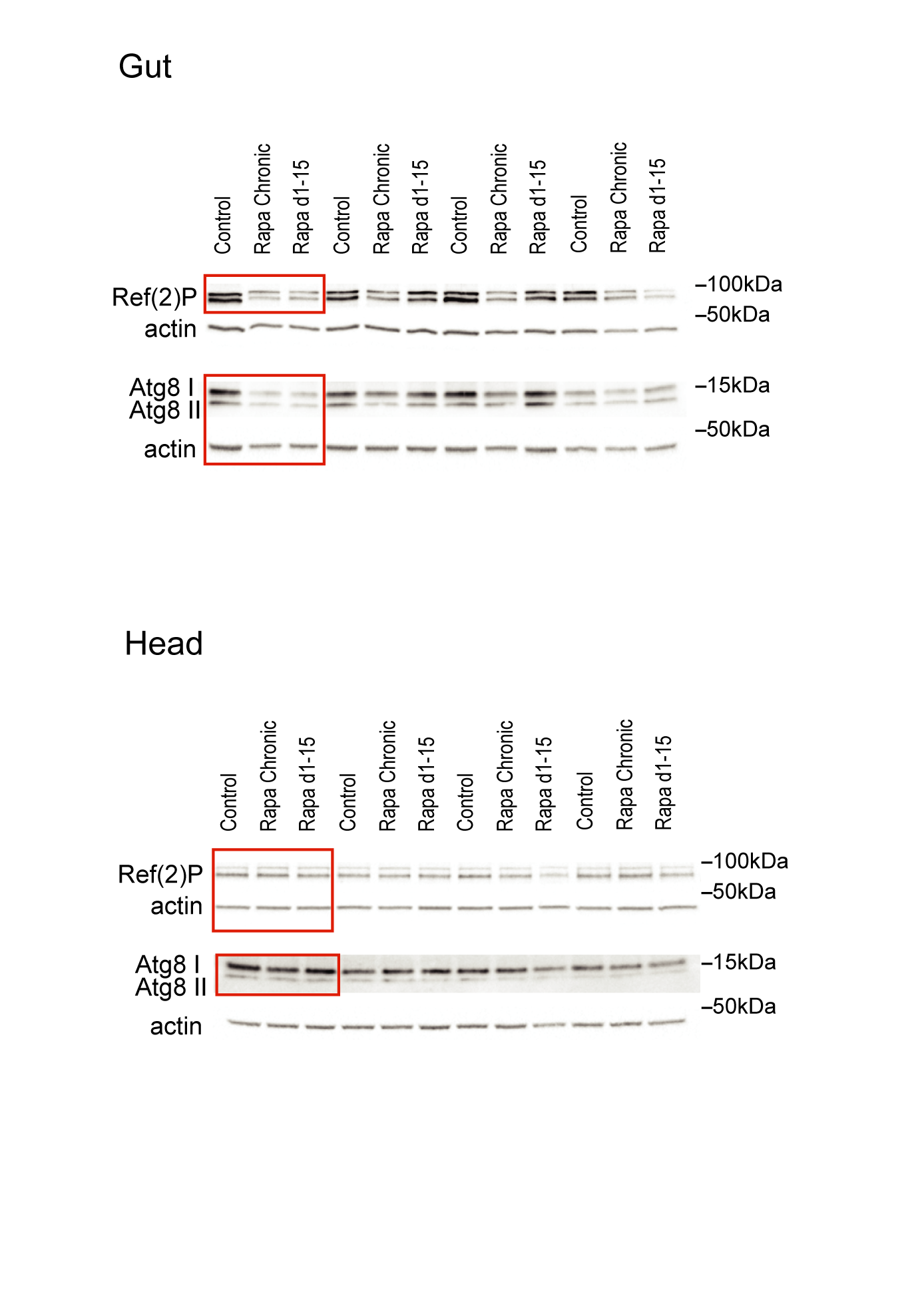

Supplement: Supplementary file 3 — Source Data Fig. 1 Unprocessed western blot for Fig. 3b,c. [file 43587_2022_278_MOESM3_ESM.tif]

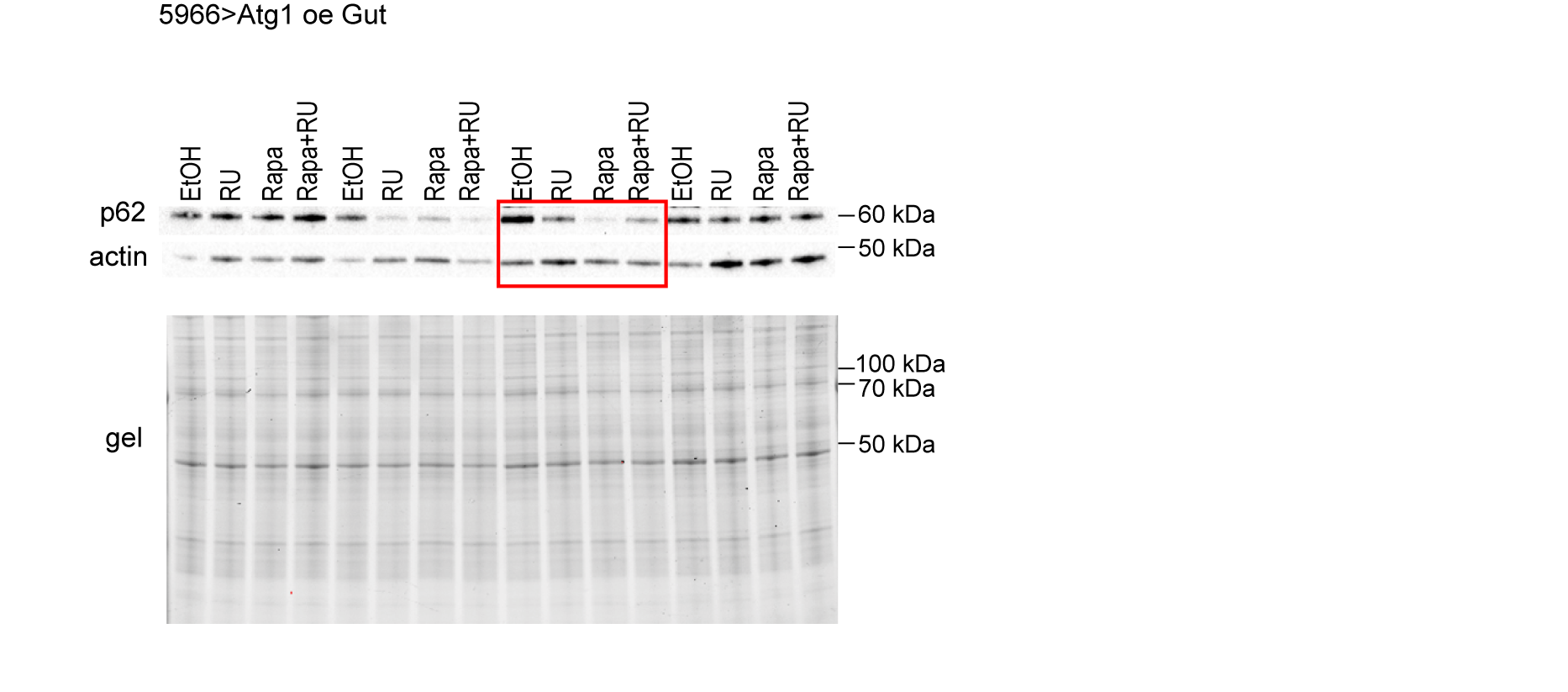

Supplement: Supplementary file 4 — Source Data Fig. 2 Unprocessed western blot for Fig. 5a. [file 43587_2022_278_MOESM4_ESM.tif]

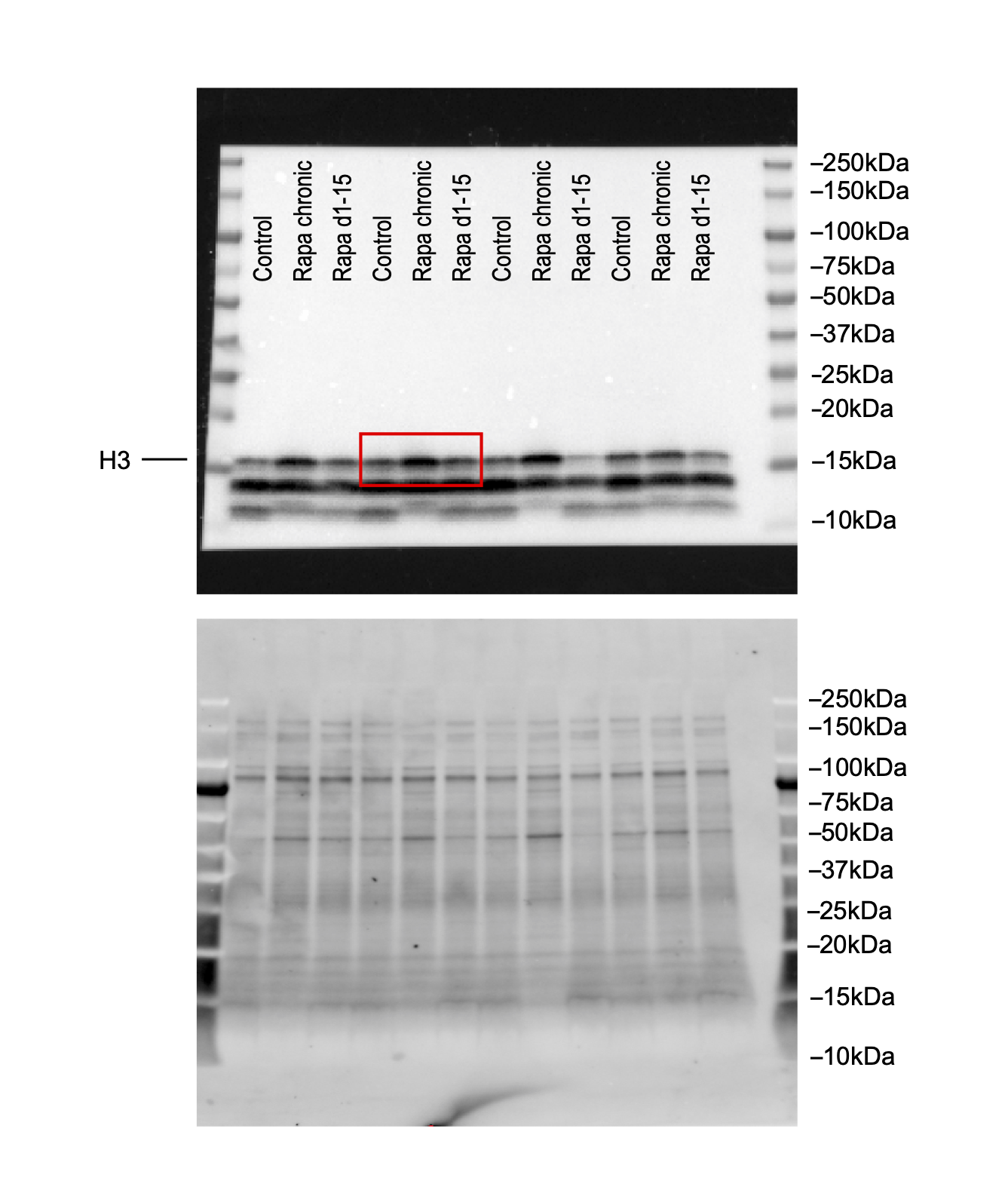

Supplement: Supplementary file 6 — Source Data Fig. 4 Unprocessed western blot for Extended Data Fig. 4a. [file 43587_2022_278_MOESM6_ESM.tif]
